# Preventing neuropathy and improving anticancer chemotherapy with a carbazole-based compound

**DOI:** 10.1126/sciadv.adw6328

**Published:** 2025-10-29

**Authors:** Lauriane Bosc, Maria Elena Pero, David Balayssac, Nathalie Jacquemot, Jordan Allard, Peggy Suzanne, Julien Vollaire, Cécile Cottet-Rousselle, Sophie Michallet, Joran Villaret, Sakina Torch, Sumari Marais, Bénédicte Elena-Herrmann, Uwe Schlattner, Anne Mercier, Véronique Josserand, Chantal Thibert, Patrick Dallemagne, Francesca Bartolini, Laurence Lafanechère

**Affiliations:** ^1^ Université Grenoble Alpes, INSERM U1209, CNRS UMR 5309, Institute for Advanced Biosciences (IAB), 38000 Grenoble, France.; ^2^ Department of Pathology and Cell Biology, Vagelos College of Physicians and Surgeons, Columbia University, New York, NY 10032, USA.; ^3^ Department of Veterinary Medicine and Animal Production, University of Naples Federico II, 80137 Naples, Italy.; ^4^ Université Clermont Auvergne, U1107, NEURO-DOL, INSERM, CHU Clermont-Ferrand, Direction de la Recherche Clinique et de l’Innovation, Clermont-Ferrand, France.; ^5^ Université Clermont Auvergne, U1107, NEURO-DOL, INSERM, Clermont-Ferrand, France.; ^6^ Université Normandie, UNICAEN, CERMN, 14032 Caen, France.; ^7^ Laboratory of Fundamental and Applied Bioenergetics, Université Grenoble Alpes, INSERM U1055, Grenoble, France.; ^8^ Department of Physiology, School of Medicine, Faculty of Health Sciences, University of Pretoria, Pretoria 0028, South Africa.; ^9^ Institut Universitaire de France (IUF), 75231 Paris, France.

## Abstract

While advances in cancer therapy have improved remission rates, chemotherapy-induced peripheral neuropathy (CIPN) remains a lasting and untreatable side effect. This study introduces Carba1, a bifunctional carbazole compound that protects against CIPN through two mechanisms. First, Carba1 interacts with tubulin, allowing for lower doses of taxanes, common chemotherapeutics known for causing CIPN, without reducing their anticancer effectiveness. Second, Carba1 activates nicotinamide phosphoribosyltransferase (NAMPT), enhancing NAD biosynthesis and boosting the metabolic resilience of neurons and Schwann cells against chemotherapy-induced damage. Carba1 shows strong neuroprotective effects in vitro against paclitaxel, cisplatin, and bortezomib toxicity and in vivo in a rat model of paclitaxel-induced neuropathy. Crucially, Carba1 does not interfere with paclitaxel’s tumor-fighting ability or promote tumor growth. Structure-activity analyses of Carba1 derivatives reveal the potential to develop compounds with dual or solely neuroprotective effects. These findings position Carba1 as a promising candidate to prevent CIPN, with potential to enhance both cancer treatment outcomes and patients’ quality of life.

## Teaser

Carba1 prevents chemo-induced neuropathy by boosting neuron resilience, without affecting cancer treatment efficacy.

## INTRODUCTION

Microtubules (MTs) are dynamic cytoskeletal filaments with crucial roles in cell division and physiology, which depend on their rapid remodeling through polymerization and depolymerization. Targeting this finely tuned process is a major therapeutic strategy in oncology [for review, see (*1*)], and drugs that interfere with MTs are key components of chemotherapies for treating carcinomas. A number of compounds bind to the tubulin-MT cytoskeleton, at different locations (*2*). They can be roughly classified into MT-destabilizers, such as vinca alkaloids, combretastatin, and colchicine, and MT-stabilizers, such as taxanes or epothilones. Among them, paclitaxel (PTX) stands out as one of the most successful chemotherapeutic agents (*3*). PTX binds to the taxane site of tubulin and stabilizes the MT lattice by strengthening tubulin contacts (*4*). At stoichiometric concentrations, PTX promotes MT assembly, whereas, at low and clinically relevant concentrations, PTX primarily suppresses MT dynamics without substantially affecting MT-polymer mass (*5*). However, MT-interfering drugs can face resistance and cause adverse effects like neutropenia, gut toxicity, alopecia, and peripheral neuropathies (*5*, *6*).

Chemotherapy-induced peripheral neuropathy (CIPN) is a debilitating adverse effect of neurotoxic anticancer drugs, affecting millions of patients annually. Characterized by distal bilateral tingling, burning, numbness, and neuropathic pain of the limbs (*6*), CIPN affects about two-thirds of patients treated with chemotherapy (*7*). CIPN can lead to reduced chemotherapy doses, thus compromising remission chances. Unlike other chemotherapy side effects, neuropathies often persist after remission in about 25% of survivors, representing an important public health concern (*8*–*10*). Now, there are no preventive strategies, and only duloxetine has shown moderate success in managing CIPN pain (*11*, *12*).

To address resistance and CIPN toxicity with PTX, we explored compounds that, when used alone, show no notable biological effects but can enhance the efficacy of PTX, enabling a reduction in PTX dosage. We previously discovered that the compound Carba1, a derivative of the carbazole series, sensitizes cells to a low, nontoxic dose of PTX (*13*). Carba1 also showed synergy with PTX in vivo in xenografted mice. It binds with low affinity to tubulin’s colchicine site, placing MTs in a favorable state for PTX binding (*13*).

In this study, we demonstrate that Carba1 synergizes with other compounds targeting the tubulin taxane site, thereby enhancing our understanding of its structural mechanism of action on tubulin. We also provide evidence that Carba1 protects neurons from CIPN, both in vitro and in a rat model. We found that the neuroprotective action of Carba1 results from the stimulation of NAD biosynthesis through activation of the enzyme nicotinamide phosphoribosyltransferase (NAMPT), a neuroprotective mechanism applicable to various types of CIPN beyond those induced by taxanes (*14*, *15*). Last, our structure-activity relationship (SAR) analysis shows that the carbazole series can be chemically modulated to prioritize neuroprotection, offering a promising therapeutic strategy to prevent CIPN.

## RESULTS

### The synergistic effect of Carba1 is specific to compounds that bind to the taxane site on tubulin

**Fig. 1.**
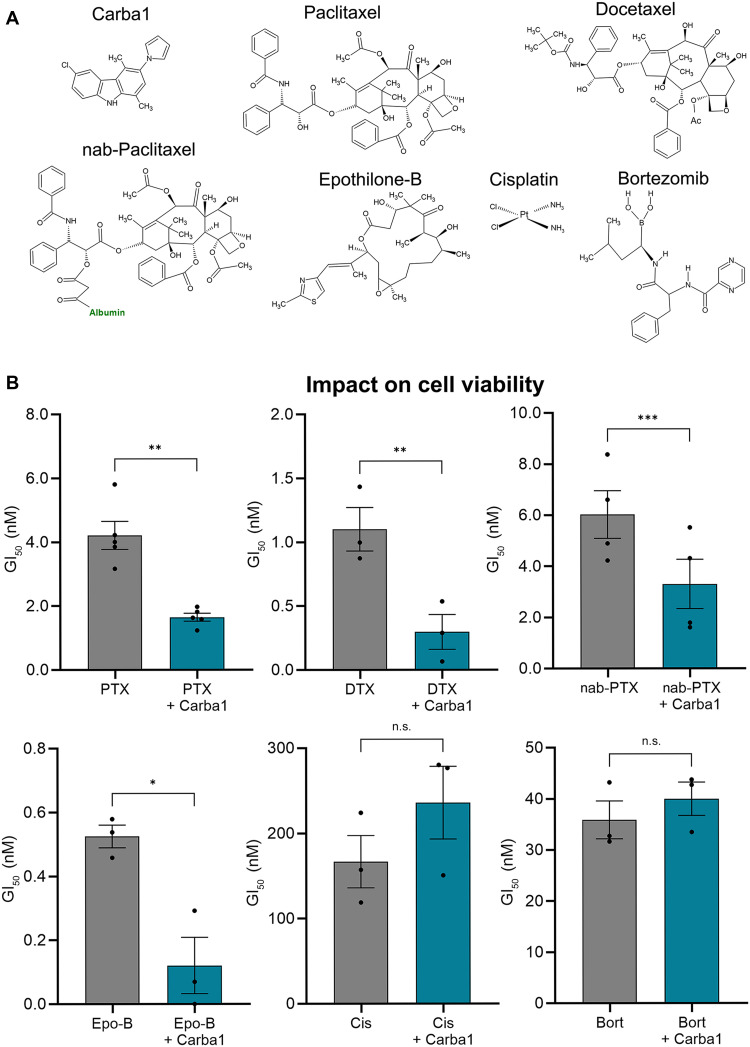
Carba1 synergizes only with chemotherapeutic agents that bind to the taxane site on tubulin. (**A**) Chemical structures of the compounds tested. (**B**) Effects of selected chemotherapeutic agents—paclitaxel (PTX), docetaxel (DTX), nab-Paclitaxel (nab-PTX), epothilone-B (Epo-B), cisplatin (Cis), and bortezomib (Bort)—on viability of HeLa cells alone or in combination with Carba1. Cells were incubated for 72 hours with the indicated concentrations of the drugs with or without 12 μM Carba1. The percentage of viable cells was calculated following a PrestoBlue assay and shown as GI_50_ of the drug when applied alone (gray) or in combination with Carba1 (blue). Data are presented as means ± SEM of at least three independent experiments.*P < 0.05; **P < 0.005; ***P < 0.0005; n.s., not significant; *t* test.

We previously demonstrated that Carba1, with minimal cytotoxicity in dividing cells with a 50% of growth inhibition (GI_50_) higher than 25 μM after a 72-hour treatment, acts in synergy with PTX (*13*). To determine whether this synergy extends to similar compounds, we analyzed the cytotoxicity induced by Carba1 in combination with docetaxel (DTX), nab-paclitaxel (nab-PTX; Abraxane), and epothilone-B (Epo-B) on HeLa cells (Fig. 1, A and B). DTX, which was originally extracted from the needles of the European yew *Taxus baccata* in the 1980s, differs from PTX, which was first extracted from the bark of the Pacific yew *Taxus brevifolia* in the 1960s. The difference lies in the 10′ position of the baccatin ring and the 3′ position of the PTX side ring. Nab-PTX is a more soluble albumin-conjugated and a recently marketed formulation of PTX, while Epo-B is a 16-membered macrolide that differs structurally from the taxanes but binds to tubulin at the taxane site (*4*). We also tested two drugs frequently used in cancer chemotherapy: the alkylating agent cisplatin (Cis) and the reversible inhibitor of the 26*S* subunit of the proteasome, bortezomib (Bort; Fig. 1A). While there is no evidence that Cis might affect tubulin, Bort has been shown to stabilize MTs in neurons (*16*, *17*) although the mechanism underlying this stabilization appears to be different from that of taxanes (*18*, *19*) and remains to be fully elucidated. As shown in Fig. 1B, we found that DTX (GI_50_ of 1.1 ± 0.2 nM) was more potent than PTX, which has a GI_50_ of 4.2 ± 0.4 nM, as previously described (*20*). On the other hand, nab-PTX was less cytotoxic, with a GI_50_ of 6.0 ± 0.9 nM. Epo-B was highly cytotoxic, with a GI_50_ of 0.5 ± 0.03 nM. The addition of 12 μM Carba1 significantly reduced the GI_50_ of all the tested drugs that impairs MTs dynamics (PTX, DTX, nab-PTX, and Epo-B by 2.6-fold, 3.8-fold, 1.8-fold, and 4.4-fold, respectively), indicating that Carba1 exerts a synergistic effect with other compounds sharing the ability to bind to the taxane site on tubulin. In contrast, the GI_50_ of Cis and Bort (166.7 ± 30.8 nM and 35.9 ± 3.7 nM, respectively) were not significantly affected when these compounds were used in combination with Carba1 (235.8 ± 42.6 nM and 40.0 ± 3.3 nM, respectively), and, instead, a slight upward trend was observed (Fig. 1B).

Together, these results indicate that the synergistic effect of Carba1 requires occupation of the tubulin taxane site, supporting our earlier findings on Carba1’s synergistic mechanism, which involves alterations in MT-lattice regions at the growing end, promoting binding of compounds to the taxane site (*13*).

### Carba1 prevents PTX-associated CIPN in vitro and in vivo

**Fig. 2.**
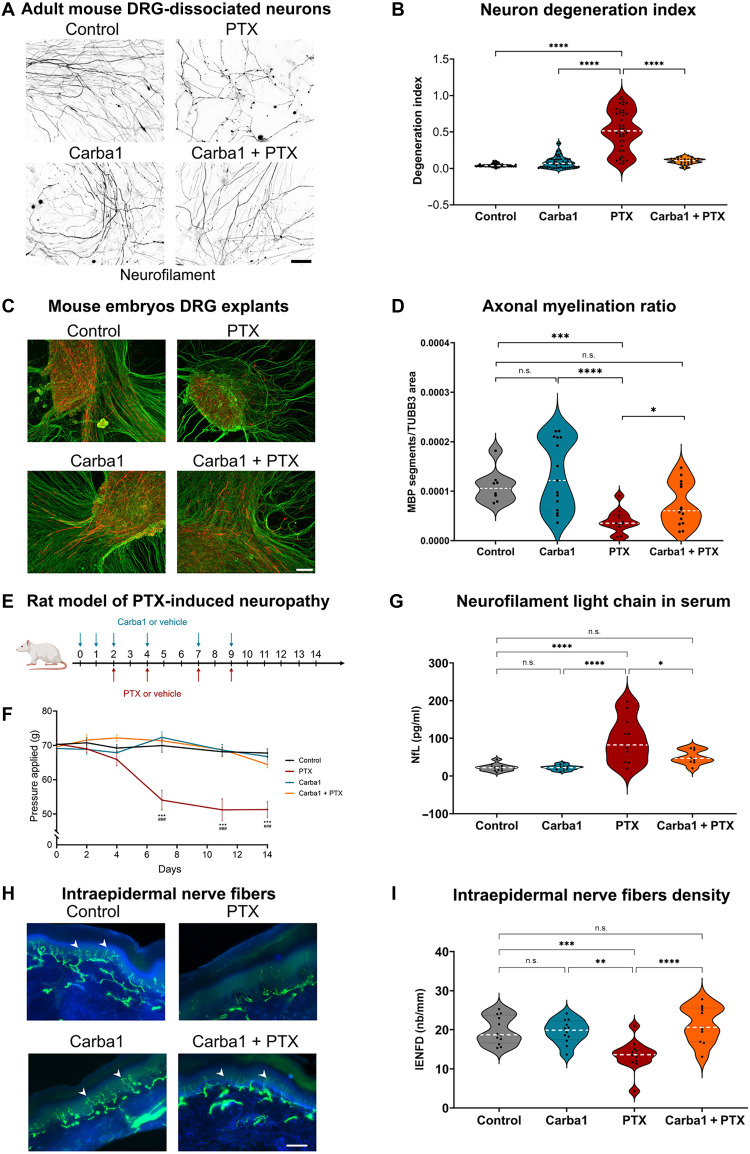
Carba1 prevents PTX-induced neuropathy in vitro and in vivo. (**A**) Representative neurofilament staining of adult mouse DRG neurons at 12 days in vitro (DIV) treated for 72 hours with dimethyl sulfoxide (DMSO; control), 50 nM PTX, 12 μM Carba1, or both. Scale bar, 50 μm. (**B**) Quantification of axonopathy using the degeneration index, calculated from neurofilament staining (see Materials and Methods and fig. S1). Data from three experiments (*n* = 16 to 55 neurites per condition for each experiment). All data points are represented as mean per field and median indicated by dashed line. ****P < 0.0001, analysis of variance (ANOVA). (**C**) DRG explants from embryonic day 13.5 mice cultured for 7 days in differentiation medium to allow Schwann cell (SC) myelination. DRG explants were treated for 72 hours with DMSO (control), 12 μM Carba1, 500 nM PTX, or both. Neurons stained with anti–beta3-tubulin (TUBB3; green) and anti–myelin basic protein (MBP; red) antibodies. Scale bar, 100 μm. (**D**) Quantification of the effect of the different treatments on myelination, represented as the ratio between the sum of the number of MBP segments and the axonal (TUBB3) area, calculated from five fields per explant (≥3 experiments). Dashed line indicates median. *P = 0.018; ***P < 0.001, Mann-Whitney test. (**E**) Experimental design for the rat model of PTX-induced neuropathy. Carba1 (50 mg/kg) was injected 2 days before PTX (5 mg/kg) and during treatment. (**F**) Carba1 reduced tactile allodynia in rats treated with PTX. Behavioral tests on days 0, 2, 4, 7, 11, and 14 (*n* = 12 per group). Means ± SEM. ***P < 0.001, PTX versus control; ###*P* < 0.001, PTX versus Carba1 and PTX, repeated-measures ANOVA with Tukey post hoc. (**G**) NfL levels in rat blood at day 15. Dashed line shows median (*n* = 12 per group). *P = 0.014; ****P < 0.0001, ANOVA. n.s., not significant. (**H**) Representative confocal images of intraepidermal nerve fibers (IENFs) from fixed rat hindpaw biopsies collected on day 15. IENFs (white arrows) were immunolabeled with PGP9.5 (green) and project from subepidermal fascicles across the epidermal-dermal junctions. Scale bar, 100 µm. (**I**) Quantification of IENF densities from hind paw biopsies. All data points are represented as single rat IENF density and the dotted white line represents the median. *n* = 12 rats per group. **P < 0.01, ***P < 0.001, ****P < 0.0001, n.s., nonsignificant; ANOVA.

We previously reported that the combined administration of Carba1 and subtherapeutic doses of PTX elicits antitumor activity in xenograft tumor-bearing mice (*13*). We posited that this combination may also mitigate the adverse effects associated with higher doses of PTX, such as those promoting CIPN. To investigate this, we directly tested the effect of Carba1 on models of PTX-associated CIPN. In patients with CIPN, a hallmark symptom is altered mechanical sensitivity resulting from the preferential damage to primary afferent long sensory nerve fibers, with secondary demyelination following axonal loss (*21*, *22*). Dying-back axonopathy is observed in both patients with CIPN and rodent models, establishing distal-to-proximal axonal peripheral nerve degeneration as a key factor underlying CIPN development (*23*). First, we examined whether Carba1 could prevent the axonal degeneration of sensory neurons (SNs) exposed to pathogenic doses of PTX (Fig. 2). To this end, primary cultures of SNs from dissociated adult mouse dorsal root ganglia (DRGs) were grown in culture for 12 days, allowing them to fully recover from axotomy and extend their axons. The cultures were then exposed to Carba1 at a dose (12 μM), which alone did not show significant effects on neurite fragmentation, with or without PTX (50 nM) administered for 72 hours. This concentration and timing of PTX treatment are widely reported to induce axonal degeneration that phenocopies the in vivo pathology (*24*, *25*). Neurons were then fixed and stained for neurofilament to assess the extent of axonal fragmentation by calculating the proportion of the total axonal area covered by the fragmented axons (fig. S1). As expected, we found that PTX induced robust axonal degeneration under these experimental conditions (Fig. 2, A and B). However, cotreatment of neurons with Carba1 and PTX prevented neuronal degeneration, as shown by the reduced number of fragmented axons (Fig. 2, A and B), indicating that Carba1 offers neuroprotection against PTX-induced axonal degeneration.

DRG explant cultures, derived from 13.5-day-old embryos, have the advantage of preserving the original architecture of the DRG, maintaining the relationships between neurons, Schwann cells (SCs), and fibroblasts (*26*). We thus examined the effect of Carba1 on PTX-induced SC injury by using ex vivo cultures of mouse DRG explants, which begin to myelinate their axons slowly from 1 week of culture, with myelination progressively intensifying toward 2 weeks of culture. We observed that 500 nM PTX was required to induce neuronal degeneration in DRG explants, most likely a result of the multicellular nature of these three-dimensional (3D) structures, which can influence the diffusion and absorption of the drug. We noticed that, under these conditions, PTX also affected the overall structure of DRGs, which appeared smaller and with a reduced axonal network density, compared to controls (Fig. 2C and fig. S2, A and C). However, when DRG explants were treated with both PTX and Carba1 (12 μM), the global structure of the DRGs and the neuronal network density were like the control group (Fig. 2C and fig. S2, A and C). Consistent with this observation, quantification of neuronal degeneration within DRG cultures, as previously conducted on isolated neurons, revealed a reduced number of fragmented axons upon PTX with Carba1 treatment, further supporting the neuroprotective effect of Carba1 also in the explants (fig. S2, A and B).

We performed myelin basic protein (MBP) staining to assess axonal myelination and found that, while Carba1 alone had no effect, PTX caused a significant reduction in MBP staining. However, when Carba1 was combined with PTX, myelin staining was substantially improved compared to PTX alone (Fig. 2C and fig. S2C). Additionally, myelin quantification determined by the number of MBP-positive segments overlapping beta3-tubulin (TUBB3)–positive areas confirmed that this increase was statistically significant (Fig. 2D).

Next, we set out to evaluate the neuroprotective efficacy of Carba1 in a rat model of CIPN. Carba1 (50 mg/kg) was injected intraperitoneally 2 days (days 0 and 1) before the initiation of PTX treatment (5 mg/kg) and repeated with each PTX injection. Rats then received either PTX, Carba1, or a combination of both on days 2, 4, 7, and 9 (Fig. 2E). The cumulative dose of PTX was 20 mg/kg and that of Carba1 was 300 mg/kg, administrated either alone or in combination. A behavioral test to assess mechanical allodynia (electronic von Frey test), characteristic of CIPN (*27*), was carried out on days 0, 2, 4, 7, 9, 11, and 14. No significant difference in the weight of the animals was observed between the different groups (fig. S2D), indicating that neither treatment had a detectable negative impact on the general health of the animals. However, rats treated with PTX developed a tactile allodynia with a significant decrease of paw withdrawal thresholds at day 7 (*P* < 0.001), in comparison to basal value at day 0, and this allodynia was still present 5 days after the last injection of PTX (Fig. 2F). There was no difference between the tactile threshold of animals treated with Carba1 alone and that of the control group, indicating that Carba1 per se was not analgesic. However, rats treated with the combination of Carba1 and PTX did not differ from the controls, and their response threshold was significantly different from that of PTX (*P* < 0.0001) (Fig. 2F), indicating that Carba1 prevents PTX-induced tactile allodynia.

We then investigated whether this protective effect of Carba1 was also detectable by histopathological analysis of intraepidermal nerve fiber density (IENFD) and serum concentration of neurofilament light chain (NfL), a biomarker of PTX-induced peripheral neuropathy (*27*, *28*). To that end, skin biopsies of hind paws collected from euthanized animals were examined for IENFD assessment and blood samples collected for NfL analysis.

We found that the NfL serum concentration was significantly increased by PTX treatment compared to control and Carba1 treatment. When Carba1 and PTX treatments are combined, however, the NfL serum concentration was significantly reduced compared to PTX treatment (Fig. 2G and table S1).

We observed that PTX also significantly reduced IENFD by 30%, whereas Carba1 had no effect and was not different from controls. However, cotreatment with Carba1 prevented PTX-induced intraepidermal nerve fiber degeneration, confirming its neuroprotective effect at the histopathological level (Fig. 2, H and I, and table S1).

### Carba1 also prevents Cis- and Bort-induced neurotoxicity in adult DRG neurons

In addition to MT-targeting anticancer drugs like PTX, several other classes of anticancer agents with distinct antineoplastic mechanisms of action can induce CIPN. We thus explored whether Carba1 could also protect against neuronal degeneration promoted by either the DNA-binding drug Cis or the proteasome inhibitor and MT-stabilizing drug Bort.

**Fig. 3.**
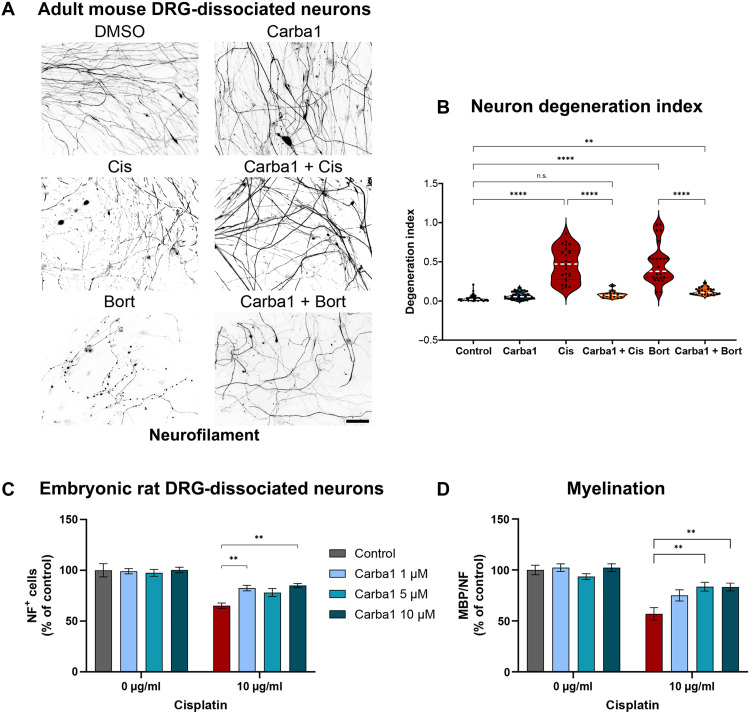
Preventive effect of Carba1 on Cis- and Bort-induced neuropathies. (**A**) Representative images of neurofilament staining in axons of adult mouse DRG neurons (DIV 12) treated for 72 hours with DMSO (control), 12 μM Carba1, 10 μM Cis, 100 nM Bort, or a combination of the drugs with Carba1 as indicated. Scale bar, 50 μm. (**B**) Quantification of the degree of axonopathy in DRG neurons treated as in (A) by degeneration index. After staining for neurofilaments, degeneration masks were generated on the basis of the method described by Gerdts *et al.* (*33*), and the index was calculated as described in fig. S1. Data are pooled from three independent experiments (eight fields per condition for each experiment). **P < 0.01; ****P < 0.0001, ANOVA. n.s., not significant. (**C**) Quantification of the effect of DMSO (control) or Cis (10 μg/ml) in combination with 1, 5, and 10 μM Carba1 on the number of rat embryonic DRG neurons. **P < 0.01, ANOVA. (**D**) Quantification of the degree of myelination in rat embryonic DRG neurons treated as in (C) and stained for MBP. **P < 0.005, ANOVA.

To this end, we compared the effect of these two anticancer drugs, with or without Carba1 on primary cultures of neurons from dissociated adult mouse DRG as we did for PTX (Fig. 2, A and B). Exposing the neurons for 72 hours to 10 μM Cis or 100 nM Bort induced significant axonal fragmentation assessed by staining the axons with the neurofilament antibody (Fig. 3A).

However, cotreatment with Carba1 (12 μM) was able to prevent neuronal fragmentation induced by either Cis or Bort (Fig. 3A). Axonal degeneration was quantified using the degeneration index (fig. S1) and showed that Cis and Bort both significantly increased the degeneration index. When Cis or Bort was coadministrated with Carba1, the value of the index was close to the control value (Fig. 3B).

To evaluate the Carba1 protective effect on neuronal myelination, we analyzed the impact of increasing doses of Carba1 on dissociated embryonic DRG neurons exposed to a toxic dose of Cis (10 μg/ml). We found that Carba1 exhibited its maximal protective effect against Cis-induced demyelination at 5 μM (Fig. 3, C and D). These results indicate that Carba1 is also able to prevent Cis- and Bort-induced neurotoxicity by protecting axonal integrity and myelination.

### Carba1 regulates energy metabolism through direct binding and activation of NAMPT

Given that Carba1 protects neurons from degeneration induced not only by PTX but also by drugs with different mechanisms of action, we hypothesized that its neuroprotective activity was due to activation of a target other than the taxanes site of tubulin, with the underlying mechanism being generally beneficial to neuronal health.

**Fig. 4.**
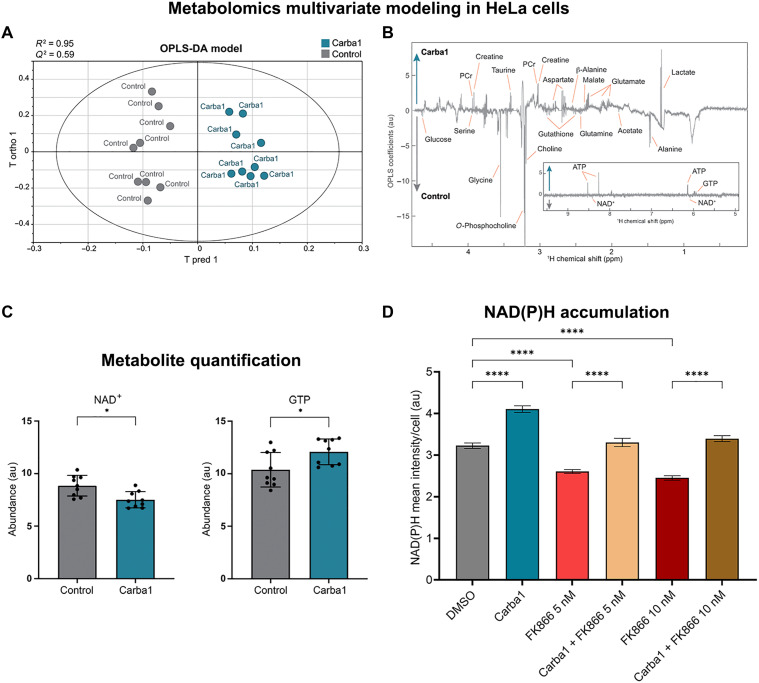
Effect of Carba1 on cell metabolism. (**A**) Score plot from OPLS-DA multivariate modeling of the NMR metabolic profiles of cells treated for 2 hours with 12 μM Carba1 (blue points) or DMSO (control, gray points). *n* = 18, with 9 replicates per sample class and 1 predictive + 3 orthogonal components; coefficient of determination for the response variable (*Y*) *R*^2^ = 0.95; cross-validated coefficient of determination for *Y* (*Q*^2^) = 0.59. (**B**) Associated OPLS-DA back-scaled loadings. Inset: Same plot, additional region [5; 9.5 parts per million (ppm)]. PCr, phosphocreatine. (**C**) Effect of Carba1 treatment on the abundance of NAD^+^ (left) and GTP (right). *P < 0.05, *t* test. (**D**) Measurement of NAD(P)H accumulation in HeLa cells. The intensity of the NAD(P)H autofluorescent signal was quantified in living cells and normalized to the cell area. Data represent the means ± SEM of three independent experiments, with a minimum of 40 cells per condition. ****P < 0.0001, ANOVA. au, arbitrary units.

Neurons are among the most energy-demanding cell types, and bioenergetic failure is considered one of the main contributors to neuronal degeneration [for review, see (*29*)]. Therefore, we first conducted an unbiased comparative metabolomic profiling of Carba1 treatment using proton-based solution nuclear magnetic resonance (^1^H NMR). Given the generality of the metabolic pathways and to gain sensitivity and reproducibility, we carried out this experiment on HeLa cells rather than isolated neurons. Aqueous extracts of HeLa cells, treated or not with 12 μM Carba1 for 2 hours were analyzed by NMR. Multivariate analyses, orthogonal projections to latent structures–discriminant analysis (OPLS-DA), clearly separated the metabolic fingerprints of each condition into two distinct groups (Fig. 4A and fig. S3). The metabolites responsible for the changes in the metabolic profile of Carba1-treated samples compared to the control are illustrated on the loading plot (Fig. 4B). Overall, this metabolic signature shows an enhanced energetic metabolism involving glycolysis (increased lactate), glutaminolysis (increased glutamate and low glutamine), and increased adenosine 5′-triphosphate (ATP), creatine, and phosphocreatine levels. Univariate statistical analysis to quantify the mean relative amplitude for each metabolite revealed a significant increase in guanosine 5′-triphosphate (GTP) levels. Such univariate analysis also uncovered a significant decrease of nicotinamide adenine dinucleotide (oxidized form) (NAD^+^) upon Carba1 treatment (Fig. 4C). These results indicate that Carba1 modulates energy metabolism. However, levels of reduced form of NAD^+^ (NADH) were below the limit of reliable quantification, preventing further estimation of the NAD^+^/NADH balance.

To find out whether Carba1 also affected cellular NADH production, we measured NAD(P)H autofluorescence following ultraviolet excitation, a minimally invasive optical approach (*30*). Live HeLa cells were incubated for 24 hours with 12 μM Carba1, and NAD(P)H intensity was monitored by confocal microscopy. We observed a significant increase of about 30% in NAD(P)H production by cells treated with Carba1 (Fig. 4D).

We posited that the metabolic changes observed (decrease in NAD^+^ and increase in NADH) could be explained by the influence of Carba1 on several molecular targets involved in the regulation of NAD^+^/NADH levels and energy metabolic pathways such as, for example, inhibition of sterile alpha and TIR motif-containing 1 enzyme (SARM1) (*31*–*33*), inhibition of poly(adenosine 5′-diphosphate–ribose) polymerase (*34*), or activation of NAMPT (*35*). NAMPT is the rate-limiting enzyme in the NAD^+^ salvage pathway that converts nicotinamide (NAM) to NAM mononucleotide (NMN), which is responsible for most of the NAD^+^ formation. We initially investigated whether NAMPT might be a target of Carba1. This was prompted by Carba1’s carbazole core, which is similar to P7C3 (fig. S4), a compound believed to target NAMPT (*36*), as well as by evidence that NAMPT activators have demonstrated neuroprotective effects (*37*, *38*). To this end, we used the potent NAMPT inhibitor FK866 (*39*), which is considered the pharmacological equivalent of genetic NAMPT silencing for NAMPT target validation (*40*).

We observed that a 24-hour incubation of cells with 5 and 10 nM FK866 was able to reduce NAD(P)H production by ~20%. When cells were preincubated with 12 μM Carba1 and then cotreated with 5 or 10 nM FK866, the levels of NAD(P)H were similar to the control levels, indicating that Carba1 was able to counteract the FK866 effect on NAD(P)H production, at both concentrations of FK866 (Fig. 4D).

**Fig. 5.**
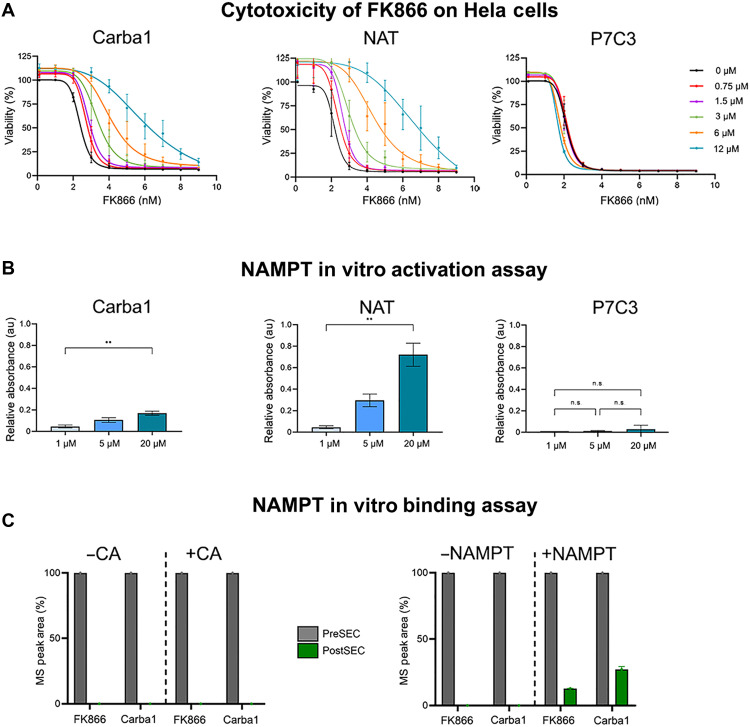
Carba1 targets NAMPT. (**A**) Carba1 and NAT, but not P7C3 relieve the cytotoxicity caused by the NAMPT inhibitor FK866 on HeLa cells. Viability dose-effect curves of FK866 in the presence of increasing doses of Carba1 (left), NAT (middle), and P7C3 (right). Data represent the means ± SEM from three independent experiments. (**B**) In vitro dose-dependent activation of NAMPT by Carba1 and NAT, but not P7C3. A triply coupled NAMPT assay was performed in the presence of the indicated doses of the compounds. Data obtained after 60 min of incubation were normalized by subtraction of their respective DMSO value, which corresponds to the basal activity of NAMPT. **P < 0.005, ANOVA. au, arbitrary units. (**C**) NAMPT binding assay using AS-MS. Carba1 (10 μM) was incubated with 3 μM NAMPT or 3 μM carbonic anhydrase (CA; negative control). FK866 (10 μM) was assayed as positive control of NAMPT binding. Protein-ligand complexes were then separated from the unbound compounds by size exclusion chromatography (SEC) and the amount of protein-bound compounds quantified by mass spectrometry (MS) after dissociation from the protein. Histograms show the amounts of compound before (preSEC; gray) and after (postSEC; green) SEC in the presence of CA or NAMPT. n.s., not significant.

FK866-induced cell depletion of NAD^+^/NAD(P)H can lead to cell death over several days. We thus investigated whether Carba1 could relieve the cytotoxicity mediated by FK866 in HeLa cells and compared Carba1 with P7C3 and the recently described potent activator of NAMPT, NAT (*37*). As shown in Fig. 5A, FK866 induced HeLa cell death, with a GI_50_ of 2.4 ± 0.09 nM. However, coadministration of Carba1 or NAT with FK866 counteracted the toxic effect of FK866 in a dose-dependent manner, inducing a shift to the right of the viability curves (GI_50_: 6.5 ± 1.3 nM for 12 μM Carba1 and 7.2 ± 2.4 nM for 12 μM NAT). P7C3 was unable to counteract the toxic effect of FK866. In contrast, Carba1’s ability to counteract FK866-induced cell death strongly suggests that, like NAT, Carba1 binds to NAMPT.

We next tested whether Carba1 could activate NAMPT in vitro. To that aim, we used a commercial recombinant human enzyme assay containing four enzymes: NAMPT, NAM nucleotide adenylyltransferase 1 (NMNAT1), alcohol dehydrogenase (ADH), and diaphorase (see Materials and Methods). In the presence of NAM and 5-phosphoribosyl-1-pyrophosphate (PRPP), NAMPT converts NAM to NMN, which is then converted into NAD^+^ by NMNAT1, while ADH converts NAD^+^ to NADH. Then, into a coupled-reaction, diaphorase converts NADH into NAD^+^ and tetrazolium salt WST-1 {4-[3-(4-iodophenyl)-2-(4-nitro-phenyl)-2H-5-tetrazolio]-1,3-benzene sulfonate} to formazan, which can be detected at optical density at 450 nm (OD_450_). In practice, we proceeded in two steps. First, Carba1 and the different tested compounds were incubated for 15 min with NAMPT, NMNAT1, NAM, and PRPP to allow NAD^+^ production. The reaction was then stopped with FK866 before adding ADH and diaphorase to convert the NAD^+^ produced to NADH, and absorbance was monitored at regular intervals. Figure 5B shows the 60-min values for Carba1, NAT, and P7C3, normalized by subtraction of their respective dimethyl sulfoxide (DMSO) value, which corresponds to the basal NAMPT activity. We found that Carba1 enhanced NAMPT activity in a dose-dependent manner, similar to NAT, although less potent. No significant effect of P7C3 was observed in this assay (Fig. 5B).

We then tested whether Carba1 directly binds to NAMPT, using affinity selection–mass spectrometry (AS-MS) (*41*, *42*). To this end, Carba1 or FK866 (positive control of NAMPT binding) was first incubated with NAMPT or carbonic anhydrase (CA; negative control). Protein-ligand complexes were separated from the unbound compounds by size exclusion chromatography (SEC). To quantify the protein-bound fraction, the complexes were then dissociated using reverse-phase chromatography, and the compounds were analyzed and quantified by mass spectrometry. By comparing the amounts of compound before (preSEC) and after (postSEC) SEC in the presence of CA or NAMPT, we assessed their ability to selectively bind to NAMPT. As shown on Fig. 5C, neither FK866 nor Carba1 was found to bind CA. However, both compounds were recovered in the fraction containing NAMPT, indicating that, like FK866, Carba1 directly binds to NAMPT.

### SAR analysis of the carbazole series enables the identification of moieties essential for the synergistic activity and those involved in the metabolic activity

Our studies indicate that Carba1 has two primary targets: It not only targets tubulin and synergizes with taxanes, but it also activates the NAMPT enzyme, thus providing neuroprotective effects in neurons affected by chemotherapeutic agents.

**Fig. 6.**
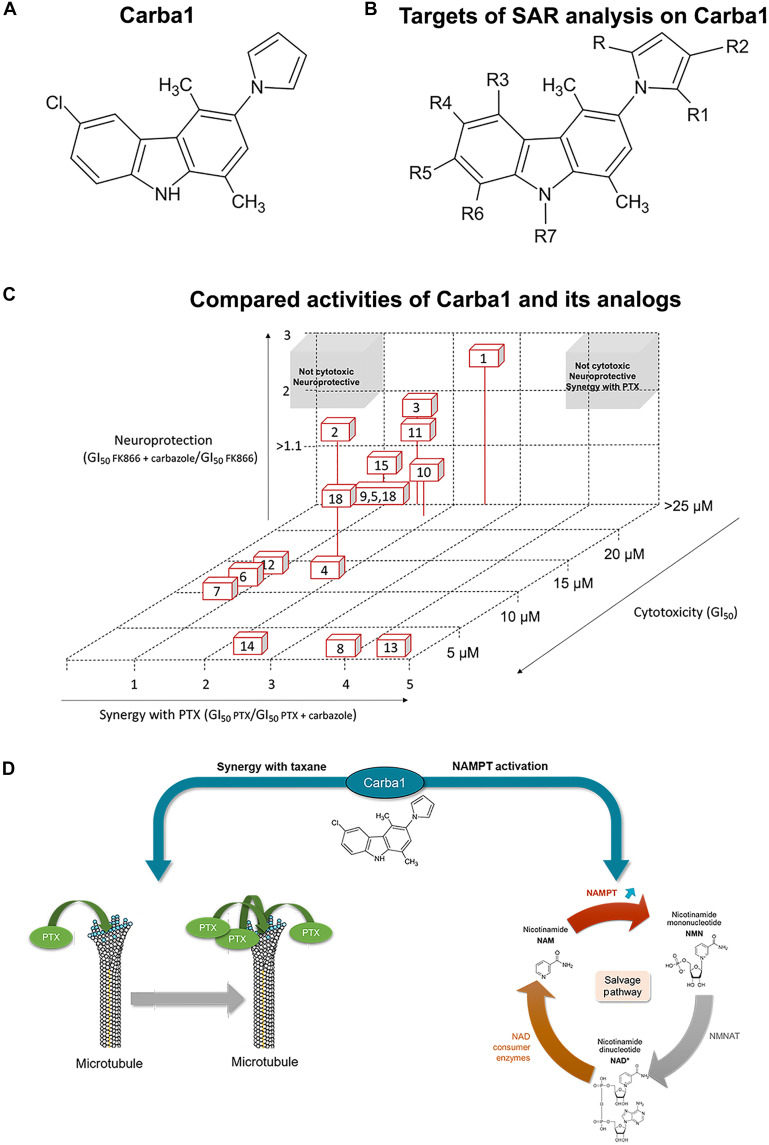
Structure activity relationship analysis of Carba1. (**A**) Carba1 chemical structure. (**B**) Positions targeted for SAR investigation. (**C**) Compared activities of Carba1 (*1*) and its analogs. (**D**) Schematic representation of the various activities of Carba1.

To gain insights into how Carba1 (Fig. 6A) interacts with these targets and whether the same chemical moieties are involved in these interactions, we conducted a SAR analysis on 16 structural analogs of Carba1. The positions of Carba1 targeted for SAR investigation are shown in Fig. 6B.

We conducted the SAR analysis by comparing the different cellular activities of these compounds. We first analyzed the effect of each compound on cell viability. We found that substituting R2, with a hydroxymethyl or a formyl group, conferred higher cytotoxicity (GI_50_ in the micromolar range) to compounds **8** and **13**, respectively, compared to Carba1 (**1**). Similarly, the substitution of R2 by a formyl group of the bromo analog of Carba1, compound **2**, induced a higher cytotoxicity to compound **14** (Table 1 and Fig. 6B). The substitution of R2 by a methylamino group also made compounds **6** and **7** more toxic than Carba1, but to a lesser extent (GI_50_ in the decamicromolar range). The same applies to the positional isomer of Carba1, compound **4** with a chlorine atom in R5, and also to the dimethylpyrrolyl analog of Carba1 (**12**). The dimethyl analog **9** of the bromo derivative **2**, however, was devoid of the cytotoxicity expressed by the latter (Table 1).

**Table 1.** SAR of Carba1 substitution.

Compound	R	R1	R2	R3	R4	R5	R6	R7	GI_50_ (μM)	GI_50_ PTX/GI_50_ PTX with carbazole^*^	GI_50_ FK866 with carbazole/GI_50_ FK866^*^
**1**	H	H	H	H	Cl	H	H	H	>25	2.5	2.6
**2**	H	H	H	H	Br	H	H	H	16	1.6	2
**3**	H	H	H	H	H	H	Et	H	>25	1.5	1.5
**4**	H	H	H	H	H	Cl	H	H	15	1.8	0.93
**5**	H	H	H	Cl	H	H	H	H	>25	1	1.07
**6**	H	H	CH_2_NMe_2_	H	Cl	H	H	H	12.7	0.8	1
**7**	H	H	CH_2_NHEt	H	Cl	H	H	H	10.3	0.77	1
**8**	H	H	CH_2_OH	H	Cl	H	H	H	1	3.8	1
**9**	Me	Me	H	H	Br	H	H	H	>25	1	0.99
**10**	H	H	H	H	OC(O)OEt	H	H	H	25	1.7	1.1
**11**	H	H	H	H	OH	H	H	H	>25	1.5	1.3
**12**	Me	Me	H	H	Cl	H	H	H	14	0.9	1.09
**13**	H	H	CHO	H	Cl	H	H	H	1	4.4	1
**14**	H	H	CHO	H	Br	H	H	H	1.2	2.5	1
**15**	Me	Me	H	H	OC(O)OEt	H	H	H	>25	1	1.1
**16**	H	H	H	H	Cl	H	H	CH_2_CH(F)CH_2_NH-3-MeOPhe	>25	1.2	0.98

* The ratios were calculated for the highest noncytotoxic dose of carbazole analogs.

Using the highest noncytotoxic dose of each compound, we quantified the potential for synergy with PTX by the ratio GI_50_ PTX/GI_50_ (compound and PTX) and the potential for activation of NAMPT by the ratio GI_50_ (compound and FK866)/GI_50_ FK866 (Table 1).

For some compounds, there is a strong correlation between the cytotoxicity of the compounds and their ability to act synergistically with PTX. The most cytotoxic compounds—**13**, **8**, and **14**—showed also the best synergistic activity with PTX. The relatively less cytotoxic compounds, **2** and **4**, are also less likely to act synergistically with PTX. Conversely, the moderately cytotoxic derivatives—**6**, **7**, and **12**—are devoid of a synergistic potential with PTX. The other most noticeable exception is Carba1, which is poorly cytotoxic (GI_50_ higher than 25 μM) while showing a good synergistic activity, allowing reduction of PTX cytotoxic doses by a factor of 2.5. Compound **10**, which carries an ethylcarbonate group instead of the chlorine atom of Carba1, is also poorly cytotoxic but still shows a good synergistic activity, although less noteworthy than that of Carba1.

Using the resistance to FK866 cytotoxic activity as a readout, only four compounds, including Carba1, were found able to activate NAMPT (ratios GI_50_ FK866 with carbazole/GI_50_ FK866 > 1.1). The presence of a halogen at R4, associated with an H at R and R1, appears to be important for the NAMPT-activating activity, because the bromo analog **2** of Carba1 is still quite neuroprotective even if it becomes more cytotoxic. A low level of NAMPT-activating activity is nevertheless retained if the halogen in R4 is replaced by a hydroxyl group (**11**) or if the halogen is removed, while an ethyl group is carried in R6 (**3**). The ethylcarbonate compound **10** is still very slightly neuroprotective, perhaps due to its hydrolysis into the hydroxyl derivative **11**. The dimethyl analog of **10**, compound **15**, is also poorly neuroprotective. All types of activity are lost when the chlorine atom of Carba1 moves from R4 to the R3 position (**5**) or when the pyrrolyl ring of **2** is further dimethylated (**9**).

As Carba1 shares a carbazole scaffold with P7C3 but differs mainly by the addition of a -CH2CH(F)CH2NH-3-MeOPhe on the central ring nitrogen (fig. S4), we substituted the hydrogen on the central ring nitrogen of Carba1 with -CH2CH(F)CH2NH-3-MeOPhe (**16**). We found that such a substitution resulted in the loss of all types of activity, underlining the crucial role of this moiety.

Last, no correlation was found between the cytotoxic/synergistic activities of the compounds and their ability to activate the NAMPT enzyme, because the more cytotoxic compounds, which are also the compounds that synergize the best with PTX (**13**, **8**, and **14**), are devoid of neuroprotective activity. This SAR study indicates that it is possible to develop compounds with either synergistic or neuroprotective activity, or both, while being devoid of cytotoxicity. Pharmacomodulations of Carba1 (**1**) will be pursued with the objective to obtain derivatives located in the top right part of Fig. 6C, or only neuroprotective compounds, located in the top left part of Fig. 6C. The diagram in Fig. 6D illustrates the distinct targets of Carba1.

### Carba1 does not interfere with tumor growth or PTX anticancer activity

As we have identified that Carba1 prevents CIPN through NAMPT activation, an important consideration is whether Carba1 could accidentally promote tumor growth or interfere with therapeutic doses of PTX in mice. Our previous in vivo experiments, designed to analyze the synergistic activity of Carba1 with low doses of PTX, ruled out the possibility that Carba1 could have a protumoral activity (*13*). Nonetheless, we herein designed an in vivo experiment to specifically test the effect of a high dose of Carba1 on tumor growth and anticancer activity when applied alone or in combination with an antitumor dose of PTX that overrides the synergistic effect of Carba1 on tumor growth inhibition. Immunodeficient nude mice were injected subcutaneously in the flank with exponentially dividing HeLa cells to induce tumors. Once the tumors were established (>120 mm^3^), mice received intravenous injections of either PTX (8 mg/kg), Carba1 (60 mg/kg), a combination of PTX (8 mg/kg) and Carba1 (60 mg/kg) or vehicle alone every 2 days for a duration of 10 days. All mice showed a slight body weight loss after the first injection regardless of the treatment, but, then, their weight increased again and reverted to normal except for one of the eight mice of the vehicle group (fig. S5).

**Fig. 7.**
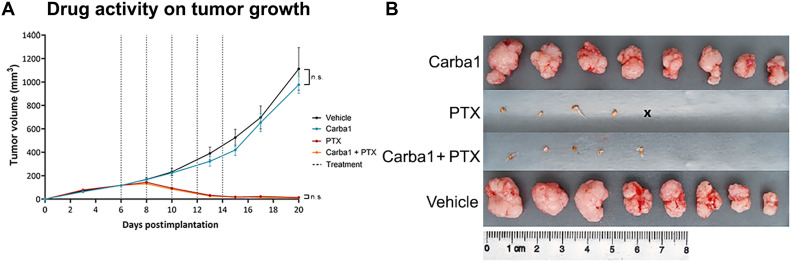
Carba1 does not affect antitumoral efficacy of a therapeutic dose of PTX. (**A**) Analysis of the effect of PTX, Carba1, and their combination on the growth of HeLa cells xenografted in mice. HeLa cells were subcutaneously implanted on the flank of female athymic nude mice. When the tumors reached a volume of about 120 mm^3^, mice were treated every 2 days (dotted lines) with PTX (8 mg/kg), Carba1 (60 mg/kg), the combination of PTX (8 mg/kg) and Carba1 (60 mg/kg), or vehicle. Tumor growth was monitored with a sliding caliper. Data are means ± SEM, *n* = 8 mice per group, ANOVA. n.s., not significant. (**B**) Images of the tumors isolated at the end of the experiment.

All clinical data analyzed in this study (blood counts and markers of renal or hepatic function) indicated that none of the treatments had any major toxicological effects (fig. S6). While we found that PTX administration markedly reduced tumor size, there were no significant tumor size changes by cotreatment with Carba1 in either vehicle-treated mice or PTX-treated mice (Fig. 7, A and B). This suggests that Carba1 neither promotes tumor growth nor inhibits the antitumor activity of an effective dose of PTX.

## DISCUSSION

Advances in cancer treatments have improved patient survival but revealed persistent side effects like CIPN. Unlike other adverse effects, CIPN can endure long term, often requiring dose reductions or treatment discontinuation, which jeopardizes clinical outcomes. While symptomatic pain relief exists, no effective strategies now prevent CIPN.

We describe Carba1, an original bifunctional agent that protects peripheral neurons from chemotherapy-induced toxicity while lowering required doses of taxanes. In vitro, Carba1 synergizes with compounds targeting the tubulin taxane site, disrupting MT dynamics to enhance the action of subsaturating doses of PTX. Additionally, Carba1 combats the neurotoxicity of CIPN-inducing agents by activating NAMPT, a key enzyme in the NAD^+^ salvage pathway, supporting a common neuroprotective mechanism (see the summary diagram in Fig. 6D).

We demonstrate in vitro that Carba1 synergizes with compounds targeting the tubulin taxane site, but not with other chemotherapeutic agents, Cis or Bort, with different mechanisms of action. This further supports the conclusion that Carba1 exerts its effect by perturbing MT dynamics at the growing end, promoting the binding of nonsaturating doses of PTX (*13*, *43*).

Although PTX, Cis, or Bort has different antitumor mechanisms of action, their main nonhematological adverse effect is CIPN.

The mechanisms responsible for the “dying-back” of distal nerve endings are still under investigation (*44*). In the case of Cis, a commonly accepted hypothesis is that the drug damages peripheral neurons through a mechanism similar to that which induces tumor cell death, namely, the accumulation of DNA-platinum adducts. This accumulation triggers apoptosis in both cancer cells and DRG neurons (*45*). Other proposed mechanisms include calpain activation and mitochondrial dysfunction, which may occur via DNA cross-linking or oxidative stress, disruption of mitochondrial motility, and alteration of the cytoskeleton (*45*–*48*).

PTX is thought to exert its neurotoxic effects through direct targeting of MTs, leading to impaired axonal transport. This disruption negatively affects cellular signaling, induces mitochondrial damage, and promotes the formation of reactive oxygen species (*49*). Similarly, Bort-induced neurotoxicity has been linked to MT hyperstabilization and the accumulation of a modified tubulin isoform, delta-2 tubulin (*50*). This modification impairs mitochondrial motility and contributes to neuronal damage (*17*).

However, although the upstream neurotoxic mechanisms may differ, they all seem to activate a common SARM1-dependent axon degeneration program that converges on axonal NAD^+^ depletion (*46*, *51*, *52*). Here, we demonstrate that Carba1 protects against the neurotoxicity of three different classes of CIPN-compounds through the binding and activation of the enzyme NAMPT, the rate-limiting enzyme in the NAD^+^ salvage pathway, supporting a common mechanism of neuroprotection.

The present study demonstrates that PTX administration at a cumulative dose of 20 mg/kg (5 mg/kg intraperitoneally, four injections) induces early signs of peripheral neuropathy in rats. These manifestations include tactile allodynia, a transient reduction in IENFD, and a modest increase in plasma NfL levels. These findings are consistent with previous studies using a lower dose of PTX (2 mg/kg, intraperitoneal), administered on a similar schedule, that resulted in prolonged tactile allodynia lasting for several weeks, up to day 93, after the final PTX injection (*53*).

However, several limitations of this model must be acknowledged. First, the relatively low dose and the intraperitoneal route of administration may limit the clinical translatability of the findings. While both intraperitoneal and intravenous routes are used in rodent models, intraperitoneal administration remains widespread due to its technical ease, lower stress for the animals, and overall better tolerability. However, it is important to acknowledge that this route does not replicate the pharmacokinetic profile of clinical intravenous administration and may thus underestimate peak systemic exposure and neurotoxicity. Although both routes are capable of producing CIPN-like symptoms, recent studies suggest that intravenous administration of higher cumulative doses is more effective in reproducing the severe, chronic phenotype observed in patients. For instance, Cavaletti *et al.* recently showed that mice receiving a very high dose of PTX (70 mg/kg, intravenous, once weekly for 4 weeks) developed persistent IENFD loss, pronounced elevation of NfL, and substantial reductions in large fiber sensory nerve conduction amplitudes, features not consistently observed with lower intraperitoneal doses (10 mg/kg, every 2 days for seven times). Of note, only with the dose of 70 mg/kg did these abnormalities persist for several weeks after treatment ended (*54*). Similarly, Meregalli *et al.* (*55*) reported plasma NfL levels exceeding 700 pg/ml after a single intravenous dose of 10 mg/kg in rats and reaching ~1500 pg/ml after 2 weekly doses, compared to 82 pg/ml in our model.

These comparisons underscore that, while our model successfully induces early features of CIPN, it may not fully replicate the pathophysiology of severe, chronic large-fiber neuropathy. Thus, this model may not recapitulate the clinical phenotype. The limited NfL elevation and IENFD changes observed here support the interpretation that our model reflects an early-stage or mild phenotype. Furthermore, the absence of electrophysiological assessments precludes conclusions regarding large fiber involvement.

Another limitation lies in the restricted behavioral evaluation. Tactile allodynia is a well-established, reproducible endpoint in CIPN models (*56*); however, it does not encompass the full range of sensory impairments seen clinically. It will be important for these results to be confirmed in additional CIPN animal models with more clinically relevant dose and administration parameters to confirm efficacy.

Despite these limitations, the combined behavioral, histological, and biomarker data offer a coherent and reproducible model of early PTX-induced neuropathy providing a foundation for assessing neuroprotective effects of Carba1 at an initial disease stage.

The neuroprotective effect of Carba1 persisted up to 14 days, indicating sustained action. High-dose Carba1 showed no detectable toxicity in mice, as confirmed by blood markers (fig. S5). While further studies are needed to establish its minimum effective and maximum tolerated doses, the results suggest that Carba1 has favorable pharmacokinetic and pharmacodynamic properties for therapeutic use, with no observed toxicity.

NAMPT activation in naïve cells after 2 hours resulted in decreased NAD^+^ and increased GTP levels, reflecting metabolic changes and feedback mechanisms to maintain NAD homeostasis. Similar studies with NAT showed notable metabolic variations at 1.5 hours that differed from those at 6 hours (*37*). Future research should focus on dynamic metabolic rewiring in naive cells, normal neurons, and PTX-stressed neurons to better understand pathways activated by PTX and Carba1’s NAMPT activation.

NAMPT overexpression is observed in various cancers, with certain types relying on NAMPT for NAD^+^ biosynthesis, making it a promising target for developing future anticancer inhibitors [for recent reviews, see Wei *et al.* (*57*), Wen *et al.* (*15*), and Velma *et al.* (*40*)]. If Carba1 or its analogs are used to prevent CIPN in the future, then it is essential to confirm that NAMPT stimulation does not promote tumor growth or compromise the anticancer efficacy of chemotherapy agents. In these experiments, a slight, nonsignificant increase in the GI_50_ of HeLa cells with Cis coadministered with Carba1 compared to Cis alone may reflect Carba1’s activation of NAMPT. Encouragingly, NMN-induced NAD^+^ elevation has shown neuroprotection against Cis-induced neurogenesis defects without affecting tumor growth or Cis antitumor efficacy (*58*). This study confirms that Carba1 does not affect the efficacy of therapeutic PTX doses and has no protumor effects, establishing it as a strong candidate for CIPN prevention.

Among the NAMPT activators reported yet (*15*), two classes of compounds, the P7C3 family with an aminopropyl carbazole core and the NAT family of hydroxyphenolamide derivatives, have been shown to prevent CIPN in animal models (*37*, *38*). While the crystal structure of the NAT-NAMPT complex has identified its binding site and activation mechanism (*37*), how P7C3 binds to and activates NAMPT remains unclear (*40*). We found that, like NAT, Carba1 could reverse cell death induced by the NAMPT inhibitor FK866, whereas P7C3 could not. Both Carba1 and NAT increased in vitro NAMPT activity, while P7C3 showed no detectable effect. These results align with previous studies that also failed to detect in vitro binding or activation of NAMPT by P7C3 (*59*–*61*). Although Carba1 shares a carbazole core with P7C3, it behaves more like NAT in its interaction with NAMPT. While we have shown that Carba1 binds directly to NAMPT through AS/MS, the crystal structure of the NAMPT-Carba1 complex is needed to fully understand the mechanism of NAMPT activation by Carba1.

Last, we compared the different cellular activities of several Carba1 chemical analogs. Although some of these molecules may be differently metabolized by the cell, this comparative analysis is indicative of the SARs of this carbazole series and relevant from a therapeutic point of view, because it is at the cellular level that drugs will ultimately act. The data suggest that compounds with either dual synergistic and neuroprotective activity or solely neuroprotective activity could be developed. However, the current data and available compounds do not allow us to predict the possibility of creating compounds with synergistic activity alone. Among the compounds studied, the one substituted on the nitrogen of the central ring (**16**) has a hybrid structure between Carba1 and P7C3 (fig. S3). This compound displayed none of the cellular activities analyzed, underscoring the differential nature of Carba1 and P7C3.

Despite these promising results, several limitations need to be taken into account when interpreting the findings and their wider implications. First, preclinical studies have been conducted mainly in vitro and in animal models, which, while informative, do not fully replicate the complexity of human CIPN or cancer biology (*62*). The long-term safety of Carba1 remains uncertain and requires further evaluation in clinical settings. These limitations underline the need for further research to validate and extend these findings, particularly through rigorous preclinical and clinical studies.

Carba1 stands out as a therapeutic candidate, uniquely combining neuroprotective efficacy through NAMPT activation with synergistic activity alongside taxanes, addressing the critical unmet need for CIPN prevention without compromising anticancer efficacy. This dual action highlights Carba1’s potential to transform chemotherapy’s therapeutic landscape, offering hope if it reaches the clinic, after regulatory preclinical development and clinical trials, for improved quality of life and treatment outcomes for patients with cancer.

## MATERIALS AND METHODS

### Study design

This study aimed to evaluate the potential of Carba1, a sensitizing agent for PTX, to enhance the efficacy of other chemotherapies, assess its neuroprotective effects against CIPN, and elucidate its mechanism of action. Carba1’s synergy with various chemotherapeutic agents was tested in vitro using viability assays on HeLa cells, while its neuroprotective effects were evaluated in vitro on dissociated neurons and DRG explants from mice and rats, and in vivo using a rat model of PTX-induced neurotoxicity. The mechanism was investigated through untargeted metabolomics, NADH imaging, NAMPT activity assays, and AS-MS binding analysis. Its impact on tumor progression and PTX’s antitumor efficacy was assessed in a HeLa cell xenograft mouse model. All procedures adhered to ethical and regulatory standards, including the National Institutes of Health (NIH) and Federation of European Laboratory Animal Science Associations (FELASA) guidelines, Animal Research: Reporting of In Vivo Experiments (ARRIVE) recommendations, and approvals from institutional and national committees (e.g., APAFIS#44532 and APAFIS#33137). Animal use was minimized, and no exclusions were made during analyses.

### Reagents

See table S2.

### Animals

The animal experiments were approved by the appropriate ethics committee. Primary cultures of dissociated adult DRG neurons were isolated from male and female C57Bl/6J mice, following protocols approved by Columbia University’s Institutional Animal Care and Use Committee in compliance with the NIH guidelines. DRG explants were cultured from C57Bl/6J embryos (harvested at 13.5 days postcoitus) following FELASA-compliant procedures at the Institute for Advanced Biosciences. In vivo studies of CIPN were conducted on 5-week-old Sprague-Dawley rats (24 males and 24 females, Janvier Labs, France), housed in standard conditions (three per cage) with ad libitum access to food and water under a 12-hour:12-hour light/dark cycle. These experiments were conducted in accordance with the ARRIVE guideline (*63*). Ethical approval for these experiments was granted by the Auvergne Animal Care and Use Committee and the French Ministry of Higher Education and Research (APAFIS#44532), with efforts to minimize animal use. Tumor growth analyses were performed on 6-week-old female Naval Medical Research Institute (NMRI) nude mice (Janvier Labs), housed at the Institute for Advanced Biosciences under ethical authorization from the Grenoble Ethics Committee (APAFIS#33137). All these animal experiments were designed to be in compliance with the 3Rs (Replacement, Reduction, Refinement).

### Preparation and staining of dissociated neurons from adult mouse DRGs

DRGs were dissected from 8- to 10-week-old mice in cold Hanks’ balanced salt solution or Dulbecco’s modified Eagle’s medium (DMEM) and dissociated in collagenase P (1 mg/ml) for 1 hour at 37°C, followed by 0.05% trypsin for 3 min at 37°C. Dissociated cells were washed with neurobasal medium supplemented with 2% B-27 Plus Supplement, 0.5 mM glutamine, 10% fetal bovine serum (FBS), and penicillin-streptomycin (100 μg/ml). Neurons were then triturated by repeated gentle pipetting until there were no more visible clumps, resuspended in neurobasal medium with 10% FBS, and plated onto 12-well plates (over Ø18-mm coverslips) that have previously been coated overnight with poly-d-lysine (100 μg/ml) at 37°C and for 1 hour at 37°C with laminin (10 μg/ml). After 4 days in vitro (DIV), at least 30% of medium was changed, and 10 μM AraC (cytosine β-d-arabino furanoside) was added to suppress nonneuronal growth. At DIV 12, neurons were treated for 72 hours with 12 μM Carba1, alone or in combination with 50 nM PTX, 10 μM Cis, or 100 nM Bort. Neurons were fixed with 4% paraformaldehyde (PFA), washed with PBS, permeabilized with PBS–0.01% Triton X-100 for 15 min, and blocked with PBS–10% FBS for 1 hour before being stained overnight at 4°C with neurofilament antibody. Following PBS washes, secondary antibody (goat anti-chicken Alexa Fluor 488) was applied for 2 hours at room temperature (R.T.). Following additional PBS washes, coverslips were mounted with Fluoromount-G.

### Preparation and staining of mouse embryonic 3D DRG explants

Embryonic DRG 3D explants were prepared from E13.5 mouse embryos and seeded at one explant/1.9 cm^2^ on acid-treated, Matrigel-coated glass coverslips (*64*, *65*). Explants were cultured at 37°C under 5% CO_2_ and high saturated in humidity. From DIV 0 to DIV 3, cultures were maintained in C-medium supplemented with 10% FBS, 1× GlutaMAX, d-glucose (4 g/liter), 2.5S nerve growth factor (NGF; 50 ng/ml), and 1% penicillin/streptomycin. From DIV 3 to DIV 7, neurobasal medium supplemented with 1× B27, 1× GlutaMAX, d-glucose (4 g/liter), 2.5S NGF (50 ng/ml), and 1% penicillin/streptomycin was used to promote axon development. From DIV 7 onward, differentiation medium [C-medium supplemented with 5 μM forskolin, ascorbic acid (50 μg/ml), and heparin (25 μg/ml)] was used to support glial cell maturation and myelination, with medium changes occurring every 2 days.

At DIV 11, explants were treated for 72 hours either with DMSO (control), 12 μM Carba1, 500 nM PTX, or a combination of both. DRGs were fixed with 10% formalin for 20 min at R.T., washed with PBS, and permeabilized for 10 min with PBS–0.1% Triton X-100. Blocking was performed using a solution of 3% bovine serum albumin (BSA), 0.1% glycine, and 5% goat preimmune serum in PBS for 2 hours at R.T. DRGs were then washed in PBS and incubated overnight at 4°C with of anti-TUBB3 (1 μg/ml) and anti-MBP (1 μg/ml). After PBS washes, secondary antibodies (goat anti-mouse Alexa Fluor 488, 1:1000, and goat anti-rabbit Alexa Fluor 647, 1:1000) were applied for 1 hour at R.T. Explants were mounted with ProLong Gold antifade reagent with 4′,6-diamidino-2-phenylindole (DAPI).

### Degeneration index measurements in mouse DRG neurons and DRG 3D explants

Images of random fields of dissociated adult DRG neurons, immunostained with antineurofilament or anti-TUBB3 antibodies, were captured using a 20× objective lens (Olympus IX81) and a Sensicam QE monochrome camera (Cooke Corporation). Degeneration masks were generated on the basis of the method described by Gerdts *et al.* (*66*). Axonal degeneration was quantified by measuring the total axonal area and fragmented axonal area in the same field, using degeneration masks as shown in fig. S1. Images were processed in Fiji using global autothresholding, binarization, and the particle analyzer module to detect fragments (20 to 10,000 pixels), as previously described (*17*). The degeneration index was calculated as the ratio of fragmented axonal area to total axonal area.

### Quantification of myelin segments in DRG explants

Images of random fields of DRG explants, fixed in 4% formaldehyde and immunostained for TUBB3 and MBP, were captured with consistent acquisition times across samples, using a Zeiss AxioImager M2 microscope, equipped with a Hamamatsu Orca R2 camera. Image analysis was performed using Fiji software. The images were thresholded using the default global autothreshold method. For each field, MBP^+^ segments were counted and normalized to the TUBB3^+^ area.

### Primary culture of rat SNs and SCs

These experiments, conducted by Neuro-Sys company (www.neuro-sys.com/) involved primary SNs and SCs cultured as previously described (*67*). On DIV 19, cultures were pretreated with Carba1 (1 to 10 μM) for 1 hour before exposure to Cis (10 μg/ml) for 24 hours in presence of Carba1. On DIV 20, the medium containing Cis was replaced with fresh medium and Carba1 for another 24 hours, ending the culture on DIV 21. Cells were fixed with ethanol/acetic acid (95%/5%) for 5 min, permeabilized, and blocked with PBS containing 0.1% saponin and 10% FBS. Cells were incubated with monoclonal anti-MBP (1:1000) and polyclonal anti-neurofilament (1:500) antibodies for 2 hours, followed by CF 488 A goat anti-mouse (1:400, Sigma-Aldrich) and Alexa Fluor 568 goat anti-rabbit (1:400, Molecular Probe) for 1 hour at R.T. Hoechst was used to stain nuclei.

Thirty images per well were acquired at ×20 magnification using ImageXpress (Molecular Devices) with identical acquisition settings, and analysis was automated with MetaXpress software. Key parameters measured included total neurite length (NF; in micrometers), total neurons (NF^+^ cells), and myelination (MBP-NF overlap, in square micrometers).

### Animal model of PTX-induced peripheral neuropathy and Carba1 administration

This protocol, adapted from (*27*), involved intraperitoneal injections of PTX (5 mg/kg) on days 2, 4, 7, and 9 for a cumulative dose of 20 mg/kg (Fig. 2E). PTX was prepared by diluting a stock solution (20 mg/ml; cremophor EL:ethanol, 1:1) to 5 mg/ml in 0.9% NaCl. Control animals received an equivalent volume of the vehicle (cremophor EL:ethanol, 1:1) diluted 1:4 in 0.9% NaCl.

Carba1 was injected intraperitoneally six times (50 mg/kg) on days 0, 1, 2, 4, 7, and 9, resulting in a cumulative dose of 300 mg/kg (Fig. 2F). For each injection, Carba1 was injected intraperitoneally at 50 mg/kg on days 0, 1, 2, 4, 7, and 9, for a cumulative dose of 300 mg/kg, using a stock solution (150 mg/ml; cremophor EL:ethanol, 1:1) diluted to 50 mg/ml in 0.9% NaCl. Control animals for Carba1 received the same volume of the vehicle [cremophor EL:ethanol (1:1) diluted 1:3 in 0.9% NaCl]. Four treatment groups were defined: control (Carba1 vehicle and PTX vehicle), Carba1 (Carba1 and PTX vehicle), PTX (Carba1 vehicle and PTX), and Carba1 and PTX. To prevent cross-exposure through feces and urine, treatment groups were randomized by cage, with each cage housing three animals.

### Assessment of nociceptive disorders (tactile allodynia)

Tactile allodynia, a common sensory disorder reported in animal models of CIPN (*56*), was assessed using an electronic von Frey test (Bioseb) (*68*). Rats were placed in plastic compartments on a wire floor and allowed to habituate for 15 min. The von Frey apparatus, with a plastic tip connected to a force transducer, was applied perpendicularly to the right hind paw, and force was gradually increased until paw withdrawal. The maximum force (in grams) causing withdrawal was recorded automatically. The average of two measurements, not differing by more than 10 g, was used as the nociceptive threshold (*68*). Rats were habituated to the compartments 3 days before the first injection, and the experimenter was blinded to treatment groups.

### Assessment of serum NfL concentration

Blood samples were collected on day 15 in vials with a clot activator, left at R.T. for up to 1 hour before centrifugation at 4100 rpm at 20°C for 10 min. Serum was then collected, aliquoted, and stored at −80°C until analysis. To reduce batch effects, serum samples were randomized on assay plates. NfL concentration was measured in the serum using commercial kits (Simple Plex Ella ProteinSimple, USA; Rat NfL Kit ProteinSimple) (*27*).

### Assessment of intraepidermal nerve fiber density

Skin samples from both hind paws were fixed in 4% PFA overnight at 4°C, followed by sucrose cryoprotection (10, 20, and 30%) at 4°C overnight. After embedding in tissue freezing medium, the samples were at −80°C. Sections (20 μm) were cut using a cryostat at −17°C, washed with PBS for 5 min, and dried. The sections were blocked in PBS/0.2% Triton X-100/1%BSA for 1 hour and then incubated overnight at R.T. with anti-PGP9.5 antibody (1:400). After washing with PBS, sections were incubated with goat anti-rabbit Alexa Fluor488 secondary antibody for 2 hours, and the nuclei were stained with DAPI. Imaging was performed by acquiring 0.5 μm *Z*-stacks and exposure times for DAPI and fluorescein isothiocyanate set to 600 ms. Quantification followed the method described by Lauria *et al.* (*69*).

### Tumor xenograft in mice

Five-week-old NMRI nude mice (Janvier Labs) were implanted with 10 × 10^6^ HeLa cells (200 μl in PBS) on the right flank. On day 6, the mean tumor volume was 120 ± 4 mm^3^. Mice were then treated every 2 days (D6, D8, D10, D12, and D14) with intravenous injections of 200 μl of Carba1, PTX, or Carba1 and PTX or vehicle (14% DMSO, 14% Tween 80, and 72% PBS). Behavior and signs of pain were monitored daily. Body weight and tumor volume were measured two to three times per week using electronic calipers, with tumor volume (in cubic millimeters) calculated as length (millimeters)/2 × width^2^ (square millimeters). Tumor growth inhibition was analyzed using GraphPad Prism with one-way analysis of variance (ANOVA) and Dunn’s multiple comparisons test for treatment versus vehicle. Mice were euthanized on day 20, when the vehicle group reached ethical limits. Blood samples were collected by intracardiac puncture in EDTA-coated tubes for differential blood counts and hemograms, outsourced to IDEXX BioAnalytics. For biochemical analysis, blood was collected in heparin-lithium tubes, and plasma was isolated by centrifugation and frozen at −80°C. Plasma was analyzed using MS Scan II (Melet Schloesing) and VET16 reagent rotors to measure markers of renal and hepatic function, metabolism, and nutritional and muscular status. Differential counts, hemograms, and biochemical data were analyzed with GraphPad Prism using Kruskal-Wallis test and Dunn’s multiple comparisons test.

### Cell lines

The human HeLa cell line was tested negative for mycoplasma contamination. HeLa cells were grown in RPMI 1640 medium supplemented with penicillin/streptomycin (100 μg/ml) and 10% FBS and maintained in a humidified incubator at 37°C and 5% CO_2_.

### Cell viability assay

Cell viability was analyzed using the colorimetric PrestoBlue assay. Cells were seeded in 96-well microplates at a density of 7500 cells per well, allowed to adhere for 24 hours, and treated for 72 hours with either DMSO or drugs at indicated concentrations. PrestoBlue (10 μl) was then added to each well, followed by a 30-min incubation. Fluorescence was measured using a FLUOstar Optima microplate reader (excitation, 544 nm; emission, 580 nm; BMG Labtech).

### NMR metabolomics analyses

HeLa cells (1 × 10^6^) were seeded in Ø100-mm cell culture dishes and allowed to adhere for 48 hours. Cells were then treated with DMSO or 12 μM Carba1 for 2 hours at 37°C and 5% CO_2_. After treatment, the medium was removed, and cells were washed with PBS at R.T. before quenching with methanol at −20°C and scrapping (*70*). Dried extracts were obtained by evaporating the solvent under gentle N_2_ flow and stored at −80°C until analysis. For NMR, extracts were resuspended in 620 μl of 100% D_2_O phosphate buffer (pH 7.4), and 550 μl was transferred into 5-mm NMR tubes. Untargeted NMR analysis was performed at 27°C on a Bruker Avance IVDr spectrometer (600 MHz, ^1^H resonance) with a conventional Broadband Inverse (BBI) probe. ^1^H metabolic profiles were acquired using a NOESY experiment with water presaturation (Bruker pulse program noesygppr1d) and a spectral width of 11,904.792 Hz. Experimental parameters included a 10-ms mixing time, 2-s acquisition time, 3-s recycle delay, and 512 scans. The 90°s pulse length was calibrated at ~11.09 μs per sample after automatic shimming and tuning. NMR free induction decays were multiplied by an exponential function corresponding to a line broadening of 0.3 Hz before Fourier transform. Spectra were phased, baseline corrected, referenced to the alanine doublet at 1.47 parts per million (ppm), and bucketed into 9900 variables (10^−3^ ppm wide) excluding water and methanol signals before multivariate statistical analysis. Data were normalized to the total sum of intensities. OPLS-DA was carried out on Pareto-scaled variables using SIMCA 17 (Sartorius). Models were validated using sevenfold cross-validation and permutation testing. Individual metabolite levels were estimated by interactive line fitting using ChenomX NMR Suite 10 (ChenomX, Edmonton, Canada), and subsequent univariate statistics were obtained using GraphPad Prism.

### Imaging and quantification of endogenous NAD(P)H production

HeLa cells were seeded in four-chamber Labtek plates at 10,000 cells per well. After 48 hours, cells were preincubated with or without 12 μM Carba1 for 2 hours before addition of FK866 (5 and 10 nM). Drugs were added to phenol red–free DMEM medium, supplemented with 10% FBS 1% penicillin/streptomycin. After 24 hours of drug incubation, cells were imaged using a confocal microscope (LSM 710, Zeiss) with a two-photon excitation at 700 nm (Chameleon Vision Laser, Coherent) and a 40×/1.2 water-immersion objective. Detection was performed in single-photon mode using an avalanche photodiode detector (ConfoCor 3). NAD(P)H signal intensity was quantified as the mean signal per cell using ImageJ and normalized to the cell area.

### NAMPT activity assay

NAMPT activity was measured using the commercial kit, NAMPT Activity Assay, according to the manufacturer’s instructions. In brief, NAMPT was incubated with various concentrations of the compounds in the presence of ATP, NAM, NMNAT1, and PRPP at 30°C for 15 min to allow the production of NAD. The reaction was stopped by adding 20 μM FK866 (NAMPT inhibitor). A mixture of water-soluble tetrazolium salts (WST-1), ADH, diaphorase, and ethanol was added and the absorbance (OD_450_) measured for 90 min using the microplate reader ClarioSTAR (BMG Labtech).

### Assay for NAMPT-compound binding

AS-MS was used to assess compound binding to NAMPT. AS-MS was performed by Edelris SAS (www.edelris.com/), which has developed the commercial AS-MS service “EDEN platform,” based on (*71*). Conditions consisted of protein (NAMPT and CA) at 3 μM and compounds (Carba1 and FK866) at 10 μM dissolved in corporate buffer.

### Statistical analysis

Each in vitro experiment was performed at least three times independently. Data shown in each figure were analyzed by appropriate statistical tests specified in the figure legends.
