# Supplementary material for: Preventing neuropathy and improving anticancer chemotherapy with a carbazole-based compound
Source: Sci Adv. 2025 Oct 29;11(44):eadw6328. doi: 10.1126/sciadv.adw6328 (PMC12571071; doi:10.1126/sciadv.adw6328)
Supplement: Supplementary file 1 — Figs. S1 to S6 Tables S1 and S2 [file sciadv.adw6328_sm.pdf]

Science Advances
AAAS

Supplementary Materials for

Preventing neuropathy and improving anticancer chemotherapy with a carbazole-based compound

Lauriane Bosc *et al.*

Corresponding author: Laurence Lafanechère, laurence.lafanechere@univ-grenoble-alpes.fr; Francesca Bartolini, fb2131@cumc.columbia.edu

Sci. Adv. **11**, eadw6328 (2025)
DOI: 10.1126/sciadv.adw6328

This PDF file includes:

Figs. S1 to S6
Tables S1 and S2

Legends of supplementary figures

Fig. S1

Representative images of isolated neurons of adult mouse DRGs treated or not with 50 nM PTX and immunostained with anti-neurofilament antibody (left). The images on the right show the degeneration masks of the same fields, generated for the calculation of the degeneration index.The red arrowheads indicate examples of degenerating axons visible in the micrograph, which correspond to the same regions highlighted in the degeneration mask.

Fig. S2

(A) Representative images of neuronal staining (TUBB3) in axons of 3D DRG mouse explants treated for 72 h with DMSO (Control), 500 nM of paclitaxel (PTX), 12 μM of Carba1 or their combination as indicated. Scale bar, 20 μm.

(B) Representative images of neuronal (TUBB3, green) and Myelin Basic Protein staining (MBP, red) from 3D DRG mouse explants treated for 72 h with DMSO (Control), 500 nM of PTX, 12 μM of Carba1or their combination as indicated. Scale bar, 100 μm.

(C) Effects of the different indicated treatments on weight gains of rats in a model of PTX induced neuropathy. Control animals (black curves) received vehicle injections. Weight of animals injected with 50 mg/kg Carba1 (blue curve), with 5 mg/kg PTX (red curve) and Carba1 together with PTX (orange curve), according to the experimental design at the indicated days is shown in Figure 2F. No significant difference (ANOVA) was observed.

Fig. S3

Validation of the OPLS-DA model of Fig 4A-B: permutation testing with 999 permutations.

Fig. S4

(A) Structure of Carba.

(B) Structure of P7C3. The common carbazole core is highlighted in orange.

Fig. S5

Weight of mice treated with PTX (8 mg/kg, red), Carba1 (60 mg/kg, blue), the combination (orange) of PTX (8 mg/kg) and Carba1 (60 mg/kg), or the vehicle (black). Dotted lines indicate treatment injection days.

Fig. S6

(A) Mouse blood cell counts

(B) Mouse renal parameters

(C) Mouse metabolic parameters

(D) Mouse liver parameter

Supp. Figure S1

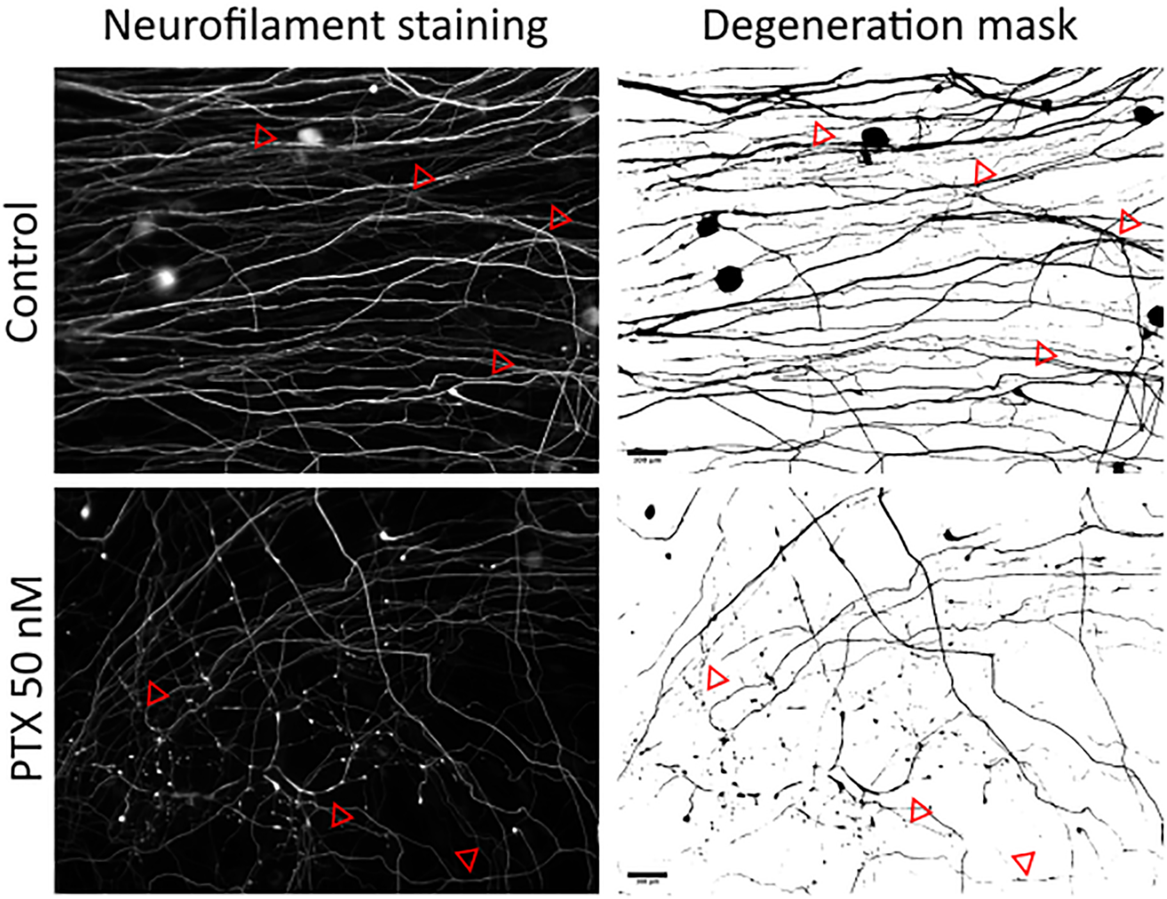

Supp. Figure S2

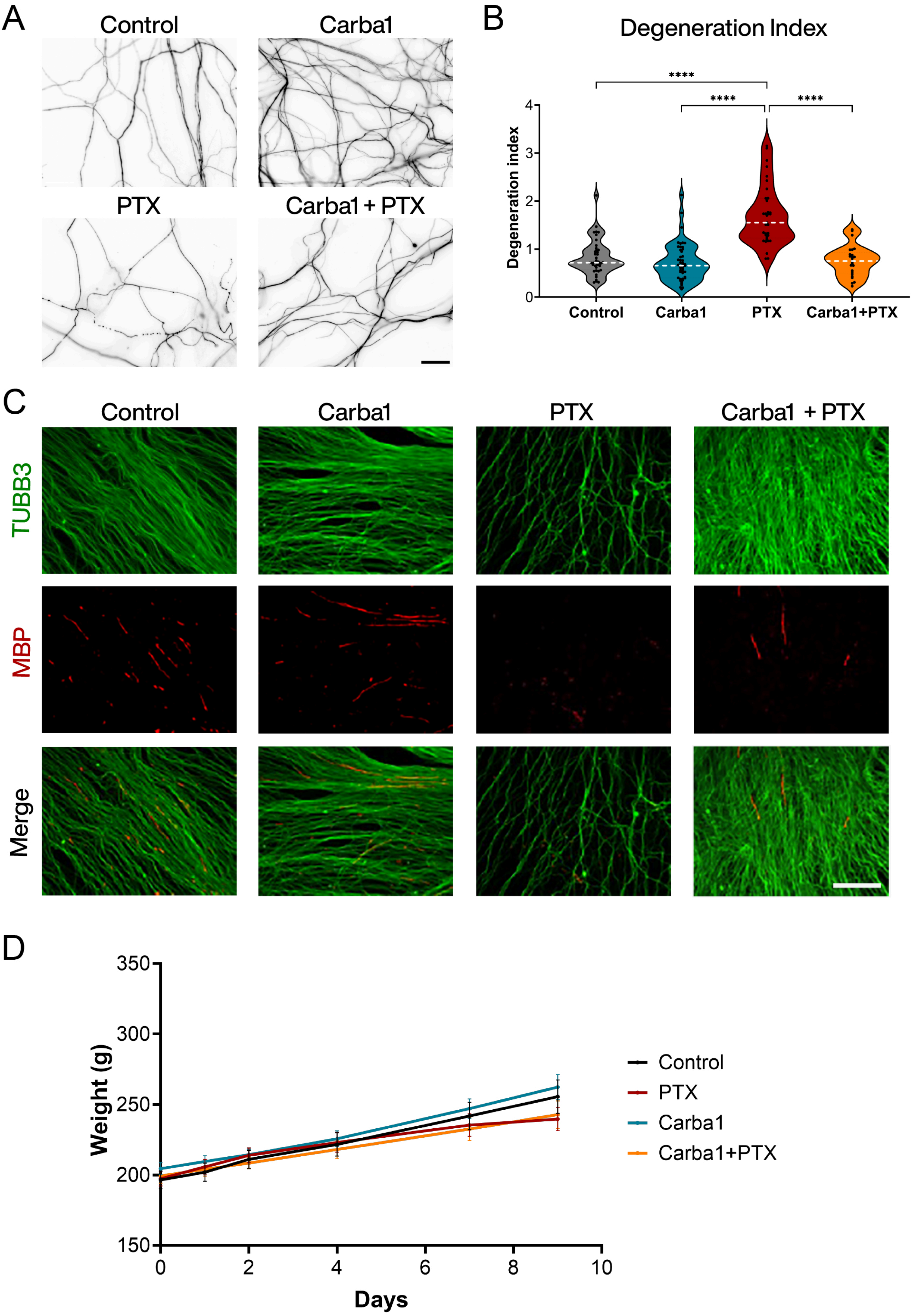

Supp. Figure 3

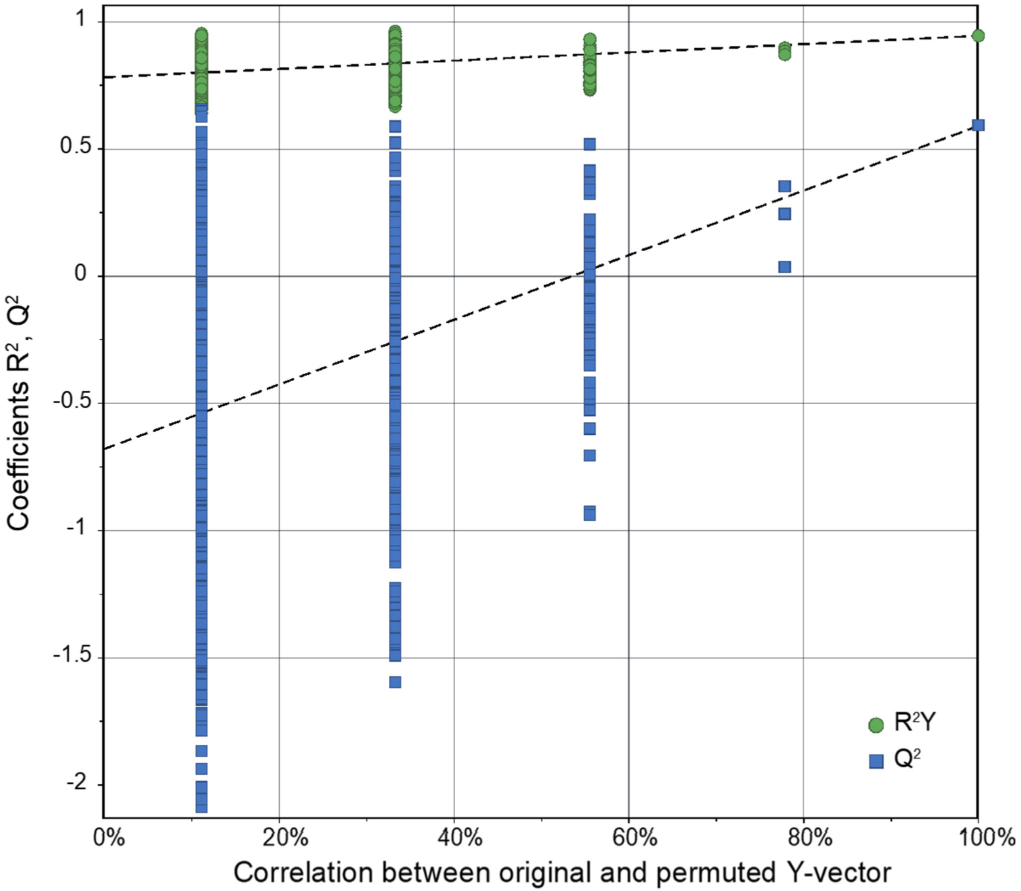

Supp. Figure 4

A Carba1

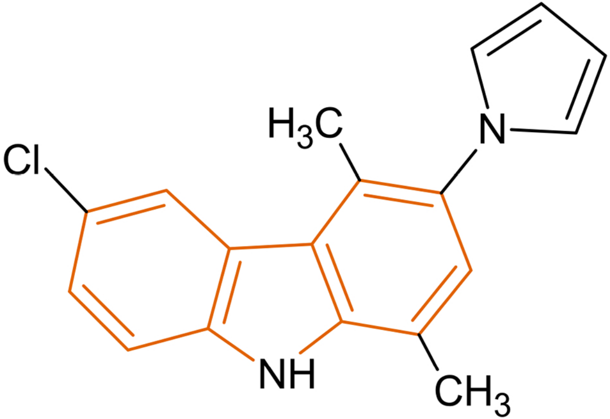

B P7C3

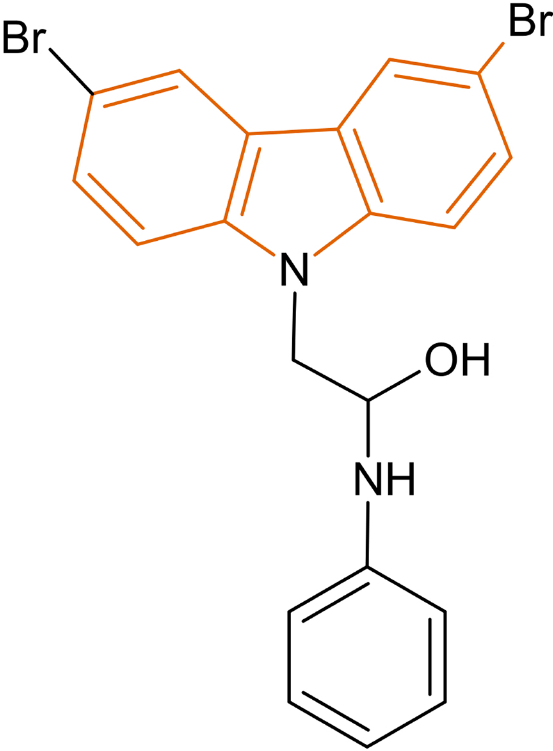

Supp. Figure 5

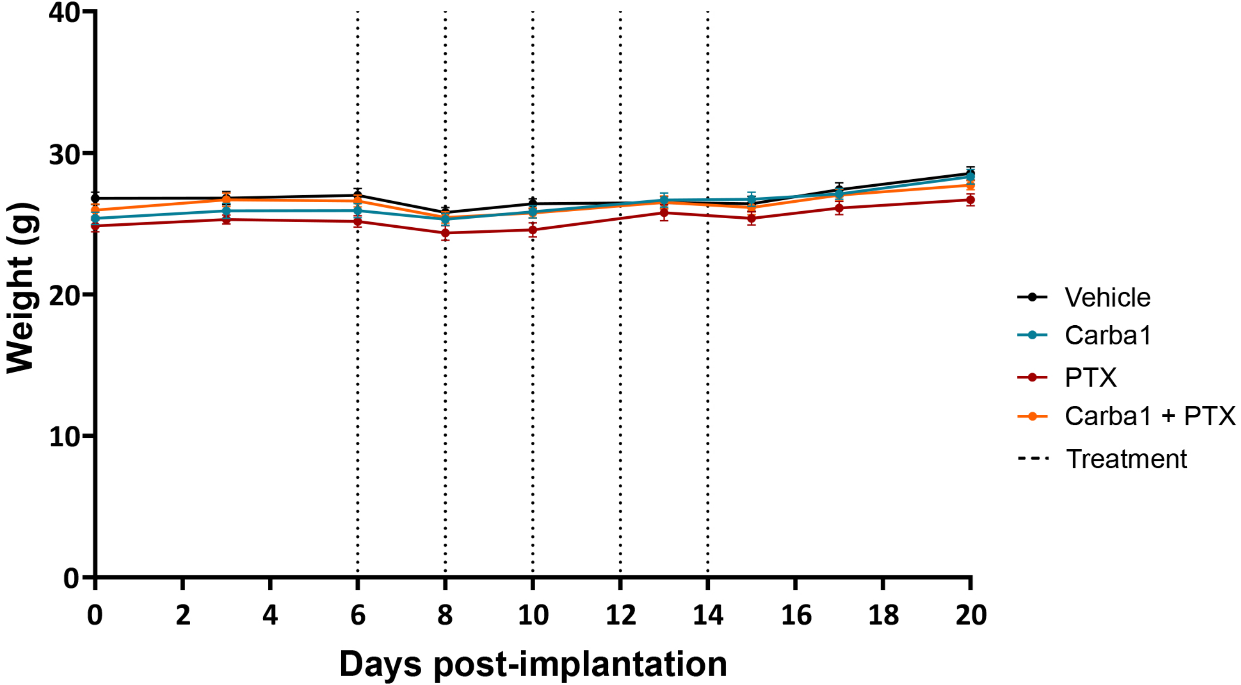

Supp. Figure 6

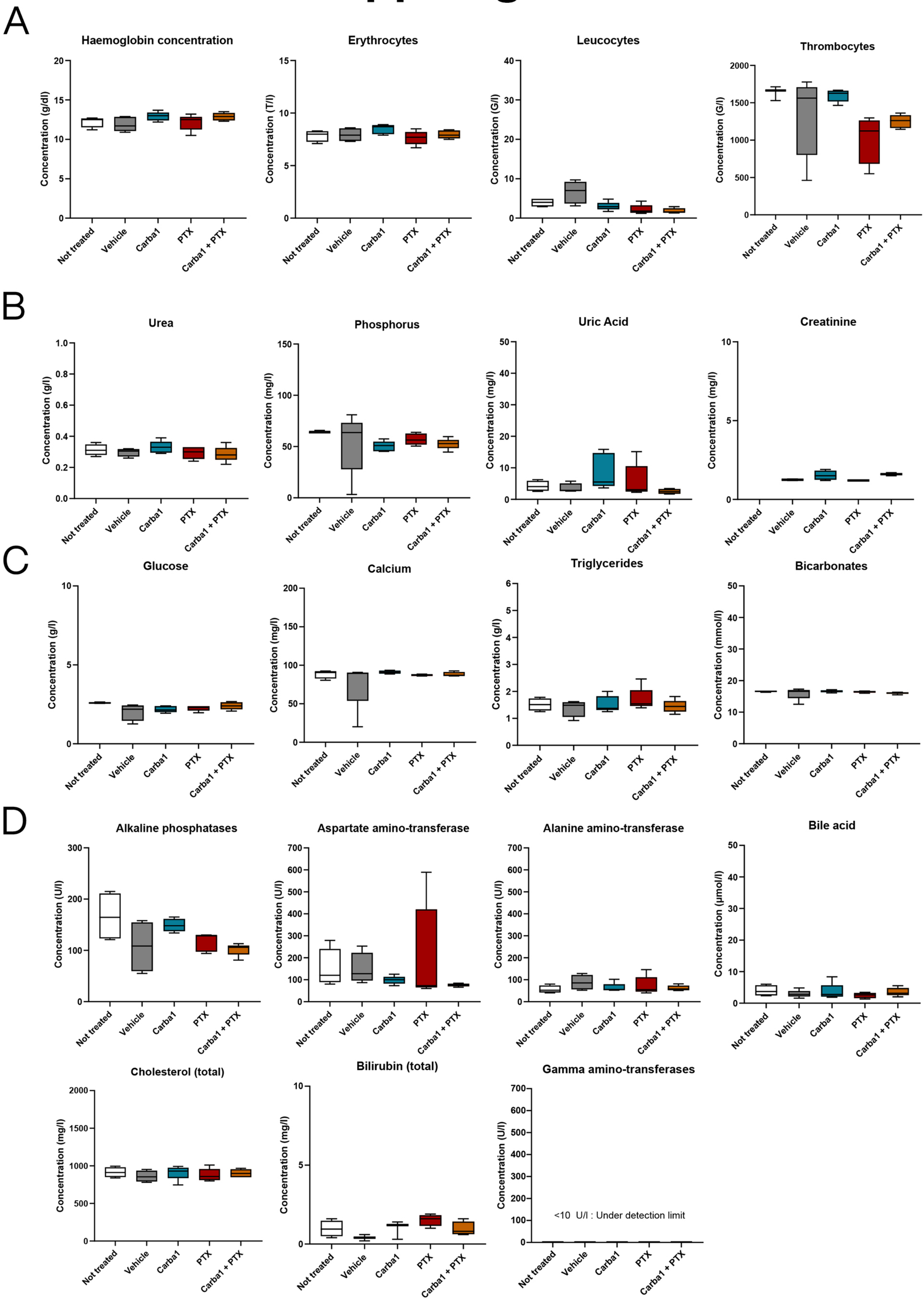

Experimental Group	NfL (pg/ml)				IENFD (number/mm)			
	Individual values	Median	Mean	SEM	Individual values	Median	Mean	SEM
**Control**	26.6<br>23.1<br>22.2<br>20.3<br>44<br>30.7<br>14.3<br>9.05<br>15.9<br>82.9*<br>24.1<br>14.3	22.20	22.23	2.88	18.9<br>18.54<br>24.22<br>15.34<br>21.42<br>22.98<br>17.61<br>25.33<br>24.04<br>15.55<br>17.95<br>16.28	18.72	19.85	1.036
**Carba1**	17.5<br>25.6<br>13.7<br>30.7<br>18<br>34.5<br>11.5<br><br>24.25<br>22.4<br>20.7<br>24.7	22.40	22.14	2.076	18.62<br>17.16<br>22.61<br>15.83<br>13.66<br>24.17<br>18.23<br>20.57<br>21.29<br>22.56<br>20.37<br>19.38	19.88	19.54	0.8764
**PTX**	143<br>82.5<br>112<br>197<br>640*<br>74.1<br>35<br>180<br>64.6<br>112<br>19.3<br>36.5	82.50	96.00	17.78	16.36<br>12<br>4.28<br>13.5<br>11.33<br>13.09<br>14.41<br>20.98<br>14.65<br>11.68<br>13.73<br>15.17	13.62	13.43	1.116
**Carba1 + PTX**	46.9<br>69.3<br><br>181*<br>41.7<br>74.9<br>250*<br>38.1<br>36.3<br>73.8<br>20.5<br>48.3	46.90	49.98	6.280	19.47<br>20.62<br><br>16.94<br>13.1<br>27.83<br>20.05<br>16.63<br>24.3<br>26.07<br>25.6<br>25.04	20.62	21.42	1.415

Table S1. Individual values and descriptive statistics for NfL and IENFD quantifications. Values indicated by * are identified as outliers (from GraphPad Prism “Identify Outliers” analysis) and removed from further analysis.

The missing measurements of both NfL and IENFD in PTX+Carba1 group is due to the death of one animal one day before the experiment endpoint.

The missing measurement in NfL for the Carba1 group was due to a problem during sample collection.

Table S2 : list of reagents, materials and antibodies used in the study

Product	Supplier	Reference
4-chamber Labtek	Dutscher	55086
96-well microplates	Greiner	655077
Anhydrous dimethyl sulfoxide (DMSO)	Sigma-Aldrich	D4540
AraC (Cytosine β-D-arabino furanoside)	Sigma-Aldrich	147-94-4
Ascorbic acid	Sigma-Aldrich	A92902
B-27 Plus Supplement	Gibco	175040441X
Bortezomib	Sigma-Aldrich	5.04314
BSA (Bovine Serum Albumin)	Sigma-Aldrich	A3912
Carba1	CERMN and Edelris	
Cisplatine (Cis)	Sigma-Aldrich	232120
Collagenase P	Sigma-Aldrich	11249002001
CremophorEL	Sigma-Aldrich	C5135
D-glucose	Gibco	A2494001
DMEM (no phenol red)	Gibco	31053028
Docetaxel (DTX)	Sigma-Aldrich	Y0001466
Dulbecco's Modified Eagle's medium (DMEM)	Life Technologies	1249015
Epothilone-B (Epo-B)	Sigma-Aldrich	E2656
Fœtal Bovine Serum	Dutscher	S1900-500C
FK866	Merck Millipore	481908
Fluormount-G	Southern Biotech	0100-01
Formalin solution	Sigma-Aldrich	HT5012

Product	Supplier	Reference
Forskolin	Sigma-Aldrich	93049
GlutaMAX™ Supplement	Invitrogen	35050061
Glycine	Euromedex	26-128-6405-C
Goat Pre-Immune serum	Gibco	16210-064
Hank's balanced salt solution (HBSS)	Life Technologies	14170112
Heparin	Sigma-Aldrich	PHR8927
Hoechst	Sigma-Aldrich	H33258
Laminin	Life Technologies	L2020
Matrigel Growth Factor Reduced	Corning	356230
MEM	Gibco	11090081
Nab-Paclitaxel	Gift from CHU Grenoble Alpes	
NAMPT Activity Assay	Abcam	ab221819
NAT	MedChemExpress	HY-144778
Neurobasal™ Medium	Gibco	21103049
Neurofilament 200kD	Aves Lab	NFH
NGF	Sigma-Aldrich	N6009
P7C3	Sigma-Aldrich	D8446
Paclitaxel (PTX)	Sigma-Aldrich	T7402
Paclitaxel (PTX, for *in vivo* rat experiments)	Leancare	
PBS	Gibco	70011044
Penicillin/Streptomycin	Gibco	15140122
PFA	Sigma-Aldrich	P6148

Product	Supplier	Reference
poly-D-lysine	Sigma-Aldrich	P1149
PrestoBlue	Invitrogen	A13262
Prolong Gold antifade reagent with DAPI	Invitrogen	P36935
RPMI 1640	Gibco	61870036
Triton X-100	Sigma-Aldrich	T8787
Trypsin	Life Technologies	25300054
**Antibodies**		
Anti-mouse AlexaFluor 488	Invitrogen	A11029
Anti-protein gene product 9.5 (PGP9.5, rabbit)	Abcam	ab15503
Goat anti-chicken AlexaFluor 488	ThermoFisher Scientific	A-11039
Goat anti-rabbit AlexaFluor 647	Invitrogen	A21245
Goat anti-rabbit AlexaFluor 488	Invitrogen	A11008
Anti-TUBB3 (mouse)	Covance	MMS-435P
Anti-MBP (rabbit)	Abcam	ab40390
